# Apoptotic bodies: bioactive treasure left behind by the dying cells with robust diagnostic and therapeutic application potentials

**DOI:** 10.1186/s12951-023-01969-1

**Published:** 2023-07-12

**Authors:** Lina Yu, Guanxiong Zhu, Zeyu Zhang, Yang Yu, Liting Zeng, Zidan Xu, Jinlong Weng, Junyi Xia, Jiang Li, Janak L. Pathak

**Affiliations:** 1 Department of Preventive Dentistry, Affiliated Stomatology Hospital of Guangzhou Medical University, Guangdong Engineering Research Center of Oral Restoration and Reconstruction, Guangzhou Key Laboratory of Basic and Applied Research of Oral Regenerative Medicine, Guangzhou, China; 2 School and Hospital of Stomatology, Guangzhou Medical University, Guangzhou, China; 3 Department of Sports and Health, Guangzhou Sport University, Guangzhou, China

**Keywords:** Apoptotic bodies, Diseases, Mesenchymal stem cells, Immunomodulation, Tissue regeneration

## Abstract

Apoptosis, a form of programmed cell death, is essential for growth and tissue homeostasis. Apoptotic bodies (ApoBDs) are a form of extracellular vesicles (EVs) released by dying cells in the last stage of apoptosis and were previously regarded as debris of dead cells. Recent studies unraveled that ApoBDs are not cell debris but the bioactive treasure left behind by the dying cells with an important role in intercellular communications related to human health and various diseases. Defective clearance of ApoBDs and infected-cells-derived ApoBDs are possible etiology of some diseases. Therefore, it is necessary to explore the function and mechanism of the action of ApoBDs in different physiological and pathological conditions. Recent advances in ApoBDs have elucidated the immunomodulatory, virus removal, vascular protection, tissue regenerative, and disease diagnostic potential of ApoBDs. Moreover, ApoBDs can be used as drug carriers enhancing drug stability, cellular uptake, and targeted therapy efficacy. These reports from the literature indicate that ApoBDs hold promising potential for diagnosis, prognosis, and treatment of various diseases, including cancer, systemic inflammatory diseases, cardiovascular diseases, and tissue regeneration. This review summarizes the recent advances in ApoBDs-related research and discusses the role of ApoBDs in health and diseases as well as the challenges and prospects of ApoBDs-based diagnostic and therapeutic applications.

**Graphical Abstract:**

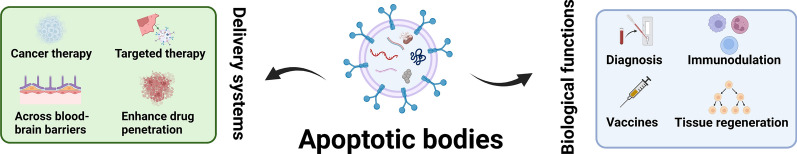

## Introduction

Apoptosis, a programmed cell death, is a natural phenomenon in all living beings and plays a crucial role in the maintenance of tissue homeostasis. The process of apoptosis includes nuclear condensation, caspase activation, DNA fragmentation, and phosphatidylserine (PS) flipping [1]. Normal and dying cells release various types of membrane-bound extracellular vesicles including microvesicles, exosomes, and ApoBDs. The nucleus and cytoplasm of apoptotic cells rapidly wrap into multiple tightly membrane-bound vesicles to form ApoBDs [2, 3]. ApoBDs are a subset of ApoBDs that are considered a tool for intercellular communication. In apoptotic death, the cell divides into various ApoBDs, which is considered a hallmark of apoptosis [4]. Subsequently, ApoBDs are engulfed by phagocytes for final degradation to prevent the release of hazardous materials in the extracellular environment [5]. ApoBDs are fragments of the apoptotic cell which are composed of externalized PS and permeable membrane, and cytoplasmic materials [6]. Although the reason for the formation of ApoBDs is unclear, it is reported that ApoBDs play a role in various diseases. ApoBDs from influenza A virus-infected monocytes can transmit the infection through the virus within ApoBDs [7]. Oxidative stress promotes the production of ApoBDs of endplate chondrocytes by inducing chondrocyte apoptosis, and oxidative stress-related ApoBDs promote mineralization of endplate chondrocytes by regulating the inorganic pyrophosphate metabolism [8]. ApoBDs also play an important role in inflammatory diseases. ApoBDs from endothelial cells carry interleukin (IL)-α and induce sterile inflammation [9]. ApoBDs from M1 macrophages promote inflammation by stimulating basal nitric oxide (NO) production [10]. In systemic lupus erythematosus (SLE), ApoBDs can be taken up by dendritic cells (DCs) to induce the maturation of DCs, which is the possible driving force of SLE [11]. In patients with lung carcinoma, the number of ApoBDs in alveolar macrophages within and close to tumor tissue can serve as a marker for malignancy [12]. These reports from the literature indicate ApoBDs as harmful debris of dead cells.

Recent reports from the literature revealed the anti-tumor, anti-inflammatory, immunomodulatory, and tissue regenerative potential of ApoBDs from different cells [2, 13–16]. It has been reported that mesenchymal stem cell (MSC) released ApoBDs enhance angiogenesis and improve myocardial infarction by regulating autophagy in endothelial cells [6]. Wang et al. reported that MSCs-derived ApoBDs inhibit tumor and inflammation in mice [14]. Therefore, ApoBDs hold a promising potential of being novel therapeutic approaches in various diseases including cancers, inflammation, and tissue defects. In addition, PS on the surface of ApoBDs functions as a “eat me” signal that triggers endocytosis by macrophage [17, 18]. ApoBDs and ApoBDs-based drug delivery systems have shown great effect in targeting drug delivery to macrophages [18, 19]. Besides, tumor cell-derived ApoBDs have great potential in the field of anti-tumor vaccines. Tumor cell-derived ApoBDs contain tumor-specific neoantigens and other tumor-associated antigens and vaccines based on tumor cell-derived ApoBDs could induce anti-tumor immunity [20, 21]. Ruben et al. reported that tumor-derived ApoBDs combined with dendritic cell-based vaccines showed the potential for the immunotherapy of acute myeloid leukemia [22]. These reports from the literature indicate the promising therapeutic potential of ApoBDs for the treatment of various diseases.

ApoBDs have potential clinical applications as disease diagnostic and prognostic biomarkers and drug carriers for the treatment of various diseases. Besides, because of the role of ApoBDs in the occurrence and development of virus infection, oxidative stress, autoimmune disease, and other diseases, modulation of contents in ApoBDs could be a meaningful strategy to treat such diseases. ApoBDs from certain cell types have shown robust potential to promote the regeneration of different tissues in the body. Based on these facts, ApoBDs can be regarded as a bioactive treasure left behind by dead cells that can be utilized as diagnostic and prognostic markers as well as therapeutic agents. This review aims to summarize the recent advances in ApoBDs-related research and discuss the prospects of ApoBDs in disease diagnosis, treatment, and tissue regeneration.

## Basic properties of ApoBDs

**Fig. 1:**
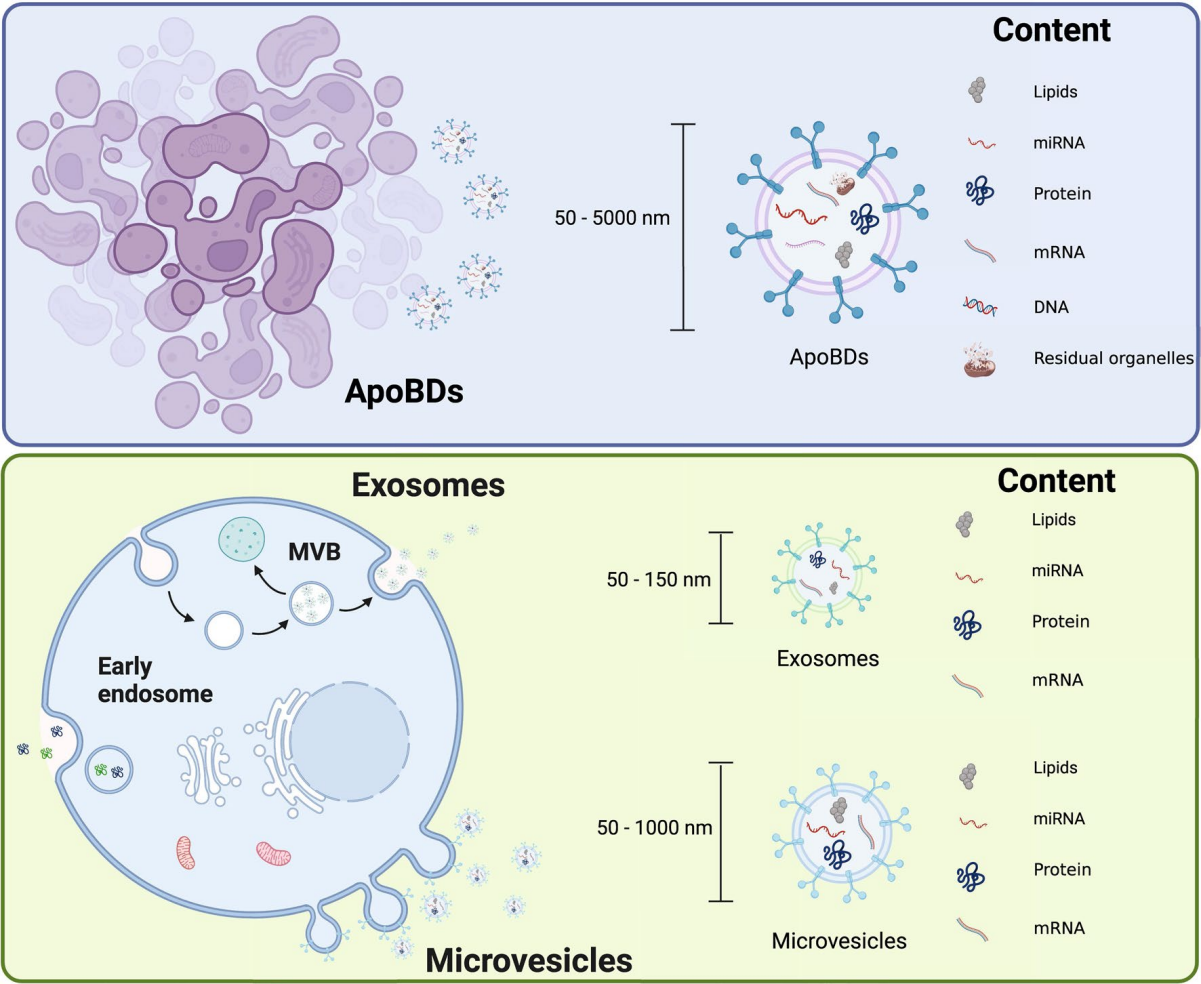
The characteristics of various EVs: exosomes, microvesicles, and ApoBDs. Created with BioRender.com

EVs, regulators of cell–cell communication, play a crucial role in providing trophic support, chemotaxis, cell proliferation and differentiation, immune responses, development, and regeneration [23–26]. EVs also participate in the pathogenesis of multiple diseases such as cancer, autoimmune diseases, and neurodegenerative disease [27–29]. Based on their sizes and biogenesis, extracellular vesicles can be broadly divided into three types: exosomes, microvesicles, and ApoBDs. Different from exosomes and microvesicles, ApoBDs are membrane-bound vesicles that are released by dying cells in the final stage of apoptosis [4]. Compared to the other extracellular vesicles, the diameter of ApoBDs is the largest (ApoBDs 50–5000 nm, exosomes 50–150 nm, microvesicles 50–1000 nm) [2, 23, 25, 30]. The morphology, lipid composition, protein markers, site of origin, mode of extracellular release, and mechanism of discharge of ApoBDs are different from the exosomes and microvesicles [31, 32]. As a new research hotspot, the only ApoBDs markers found to date are PS [33]. However, some extracellular vesicles are also characterized by externalized PS such as adipocyte-derived large extracellular vesicles [34, 35]. So, the markers of ApoBDs remain controversial. Although ApoBDs are still poorly studied, it has been proposed that the cells undergoing programmed death disintegrate into ApoBDs to contribute to more efficient clearance of apoptotic cells, intercellular communication, and immune regulation (Fig. 1) [36].

**Fig. 2:**
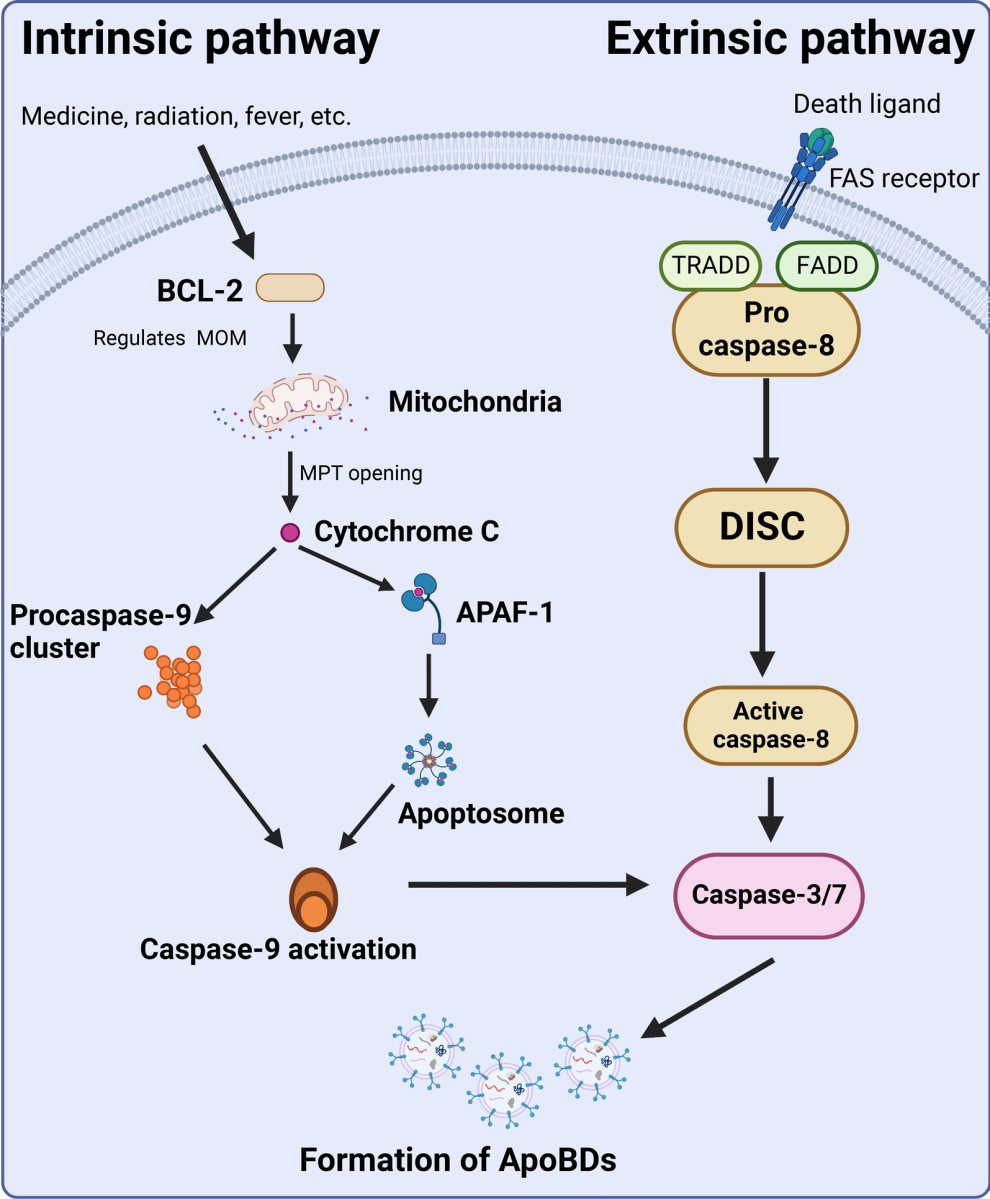
Pathways involved in apoptosis. The intrinsic pathway is activated by various stresses, such as medicine, radiation, and fever. This induces the BCL-2 protein to regulate the permeability of the mitochondrial outer membrane (MOM) and then opens the mitochondrial permeability transition pore. Cytochrome C, via binding to APAF1, causes the formation of the apoptosome, and the procaspase 9 in this process cluster. Caspase 9 proteolytically activates the effector caspases 3 and 7. The extrinsic pathway is death-receptor-induced apoptosis. FAS ligand bind to FAS receptors to activate downstream signaling. This leads to the recruitment of pro-caspase 8, FADD, and TRADD, resulting in the formation of DISC. DISC catalyzes the activation of caspase 8. Caspase 8 directly or indirectly activates caspases 3 and 7. Finally, ApoBDs are formed. Created with BioRender.com

Apoptosis can be initiated through intrinsic and extrinsic pathways. The intrinsic pathway is controlled by the BCL-2 protein family by regulating the permeability of the mitochondrial outer membrane [37]. Bax and Bak are members of the Bcl-2 family and core regulators of the intrinsic pathway of apoptosis. BAX and BAK oligomerize and permeate in the mitochondrial outer membrane to open the mitochondrial permeability transition pore which releases the intermembrane component of caspase and activates apoptosis [37, 38]. There are two main groups of intrinsic pathways. The first group consists of cytochrome C, Smac/DIABLO, and the serine protease HtrA2/Omi [38]. Cytochrome C activates the mitochondrial pathway, via binding to apoptotic peptidase activating factor 1 (APAF1), causing the formation of the apoptosome [39–41]. Then procaspase-9 reactivates caspase-9 activation. Extrinsic apoptotic pathways initiate apoptosis involving transmembrane receptor-mediated interactions. Members of the TNF receptor family play a key role in transmitting death signals from the cell surface to intracellular signaling pathways, which are called the “death domain” [41]. The binding of the FAS ligand to the FAS receptor results in the binding of the adapter protein FADD and the binding of the TNF ligand to the TNF receptor results in the binding of the adapter protein TRADD, resulting in the formation of the so-called death-inducing signaling complex (DISC) [42, 43]. DISC catalyzes the activation of caspase-8, which triggers the execution phase of apoptosis [38, 44]. Finally, the caspases produced by intrinsic and extrinsic pathways activate caspase-3/7 and complete the whole process of apoptosis (Fig. 2) [38].

The rupture of apoptotic cells into ApoBDs was previously thought to be a random process, but recent studies have shown that the formation of ApoBDs is a coordinated result. Evidence suggests that actin-myosin plays a leading role in apoptosis remodeling, the critical medium of disintegration of apoptotic cells is plasma membrane blistering [45], and membrane blebbing forms as a result of increased intracellular hydrostatic pressure following actomyosin-mediated contractions [46]. The repeated process of foaming and contracting apoptotic cells leads to the formation of ApoBDs containing organelles and other cell contents [47]. Subsequent separation of vesicles to produce ApoBDs depends on the formation of membrane prominences [48, 49]. It is important to note that apoptotic vesicles differ from necrotic vesicles, which are usually larger and unrelated to actomyosin contraction [50, 51]. Another phenomenon called apoptotic volume decrease (AVD) is also related to apoptotic body formation, and inhibition of cytoskeletal destruction of AVD prevents the formation of ApoBDs [52, 53]. AVD is an early event that occurs in conjunction with membrane blistering, leading to contractions of apoptotic cells. Different cell types exhibit different forms of membrane deformation, including microtubule spikes, apoptosis, and bead cell apoptosis, and among them, bead cell apoptosis appears to be the most efficient way to produce ApoBDs, producing approximately 10–20 ApoBDs at the same time. Bead cell apoptosis represents a unique mechanism for rapid ApoBD formation [33].

**Fig. 3:**
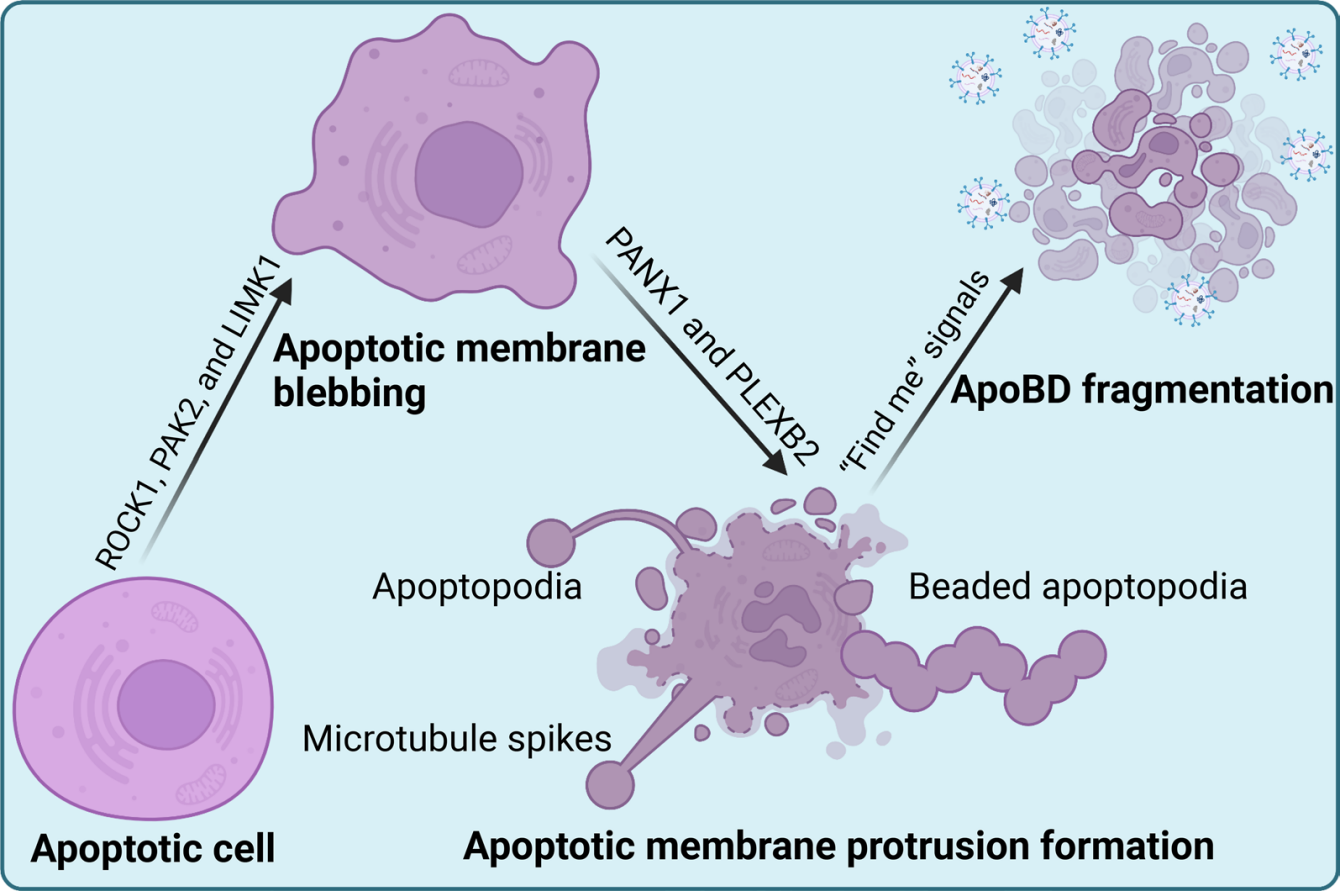
The formation of ApoBDs. First, the rupture of the cytoskeletal plasma membrane causes apoptotic membrane blebbing and the loss of the phospholipid asymmetry in the plasma membrane triggers membrane blebbing, which is thought to be regulated by kinases including the PAK2, LIMK1, and ROCK1. And then, the apoptotic membrane protrudes in different types, including microtubule spikes, apoptopodia, and beaded apoptopodia. PANX1 and PlexB2 have a major influence on their nuclear contents and size. At the later stage of cell death, the apoptotic cells are fragmented and ApoBDs are formed. Created with BioRender.com

There are three stages of ApoBDs formation. The first step is crescent-shaped spaces, which are found around the nucleus and are known as apoptotic membrane blebbing [54]. Apoptotic membrane blebbing is caused by the rupture of the cytoskeletal plasma membrane. The loss of the phospholipid asymmetry in the plasma membrane triggers membrane blebbing. The hydrostatic pressure within the cell promotes the formation of membrane blebbing, while the actin cortex acts locally to promote membrane blebbing [46, 55]. At the molecular level, apoptotic membrane blebbing is thought to be regulated by kinases including the serine/threonine caspase-activated kinases p21 activated kinase 2 (PAK2), Lim domain kinase 1 (LIMK1), and Rho-associated kinase 1 (ROCK1) [56]. The ROCK1 is considered the base for promoting apoptotic membrane blebbing, and the ROCK inhibitor Y-27632 inhibits the formation of membrane blebs [57]. At the stage of vesicle formation in the early stage of apoptosis, the active caspase 3 protein hydrolyzes ROCK1 and triggers kinase activation by releasing its self-inhibiting C-terminal domain. In addition, ROCK1 phosphorylates the myosin light chain of myosin II and promotes actomyosin contraction, which promotes membrane blister [58, 59]. The enhanced activity of ROCK1 leads to the physical destruction of the nuclear membrane and the degradation of Lamin A [55]. LIMK1 inactivates cofilin, and caspase-activated LIMK1 may support the foaming of apoptotic membranes by inhibiting cofilin and promoting actin polymerization [60, 61]. Then the nucleus invaginates moderately and blebs appear. The next step is apoptotic membrane protrusions, including microtubule spikes, apoptopodia, and beaded apoptopodia. Some studies have shown that pannexin 1 (PANX1) and PlexB2 have a major influence on apoptotic cells’ nuclear contents and size. In this step, f-actin and microtubules in a subset of apoptopodia generated by T cells and monocytes affect apoptopodia formation, while this phenomenon has not been observed in other cells [62, 63]. PANX1 has been identified as a key regulator of apoptosis formation. PANX1 is activated by Caspase 3 and 7, leading to the release of “find me” signals into extracellular space, promoting apoptosis and ApoBDs formation [49, 64–66]. The third step is nuclear blebs fragments. In this step, the nuclear fragments are compacted into half-moon shapes and then ApoBDs were formed. Among these stages, the upstream disassembly steps impact the number of ApoBDs (Fig. 3) [54, 56].

## Separation and preparation process of ApoBDs

There are three important processes to obtaining the ApoBDs. One is the induction and verification of the ApoBDs, the other is the isolation of the ApoBDs, and the third is the purification of the ApoBDs. There are several ways to induce apoptosis, including drugs, UV exposure, starvation, and hypoxia that increase the yield of ApoBDs, and each technique has its advantages and disadvantages [67–69]. However, it is still difficult to isolate and quantify ApoBDs after induction of apoptosis. The traditional method separates ApoBDs by differential centrifugation according to the density of different ApoBDs, but the purity of ApoBDs obtained by this method is relatively low [70]. Flow cytometry commonly detects Annexin V (Ca^2+^-dependent phospholipid-binding protein) and nucleic acid stain propidium iodide, which is used to detect cell apoptosis. At present, more than 90% of pure ApoBDs can be obtained by the combination of differential centrifugation and flow cytometry [71].

### Apoptosis induction in vitro for ApoBDs isolation

The number of ApoBDs produced by healthy cells is not high, so scholars use physical and chemical methods to promote cell apoptosis rate and collect more ApoBDs for research and clinical applications. Currently, the two most commonly used methods of inducing apoptosis include treating cells with 0.5 μM STS for 3–12 h [6, 72] and UV (150 mJ/cm^2^) exposure [33, 73, 74]. The protein kinase inhibitor STS is one of the most effective and commonly used pro-apoptotic stimulants, although its mechanism of action is poorly understood. Some scholars have pointed out that STS-induced apoptosis of cardiomyocytes and human corneal endothelial cells involves the activation of caspase, mainly caspase-3 [75, 76]. However, whether other cells have the same characteristics needs to be confirmed. STS induces apoptosis through both caspase-dependent and caspase-independent mechanisms [77]. In addition, STS induces caspase-9 activation and apoptosis without APAF1 [78]. Recent studies have shown that AVD is not entirely induced by STS, but depends on cell type and substrate attachment [79]. Another commonly used method of inducing apoptosis is UV exposure, which induces apoptosis with different effects than drugs. It has been reported that UV-irradiated Jurkat cells exhibit typical apoptotic symptoms: morphological changes, internucleosomal and high molecular weight DNA fragmentation, G1 sublevel formation in the frequency histogram of DNA content, and dissipation of mitochondrial transmembrane potential [80]. Further experiments showed that caspase was inhibited internally and externally during UV-induced Jurkat cell death [81]. Yuan et al. induced endplate chondrocyte apoptosis with H_2_O_2_ (1 mmol/L) for 12 h and obtained ApoBDs [8]. Other methods include induction by 500 μM azbisphosphonate alendronate for 24 h at 37 ℃, serum-free culture, and anti-Fas treatment (250 ng ml−1) [33, 82]. Berda-haddad et al. tried serum starvation combined with TNF-α stimulation to induce human umbilical vein endothelial cells apoptosis [9]. For the induction of apoptosis, the Jurkat cells were stimulated with either etoposide (10 µM), actinomycin-D(5 µg/ml), TNFα (100 µM), or STS (10 µM) for 24 h, heated at 56 ℃ for 30 min in a water bath or irradiation with a UV light source for 10 min at a distance of 10 cm [83]. The results showed that the drug induces apoptosis at the highest level, followed by UV irradiation, and the water bath induces apoptosis at the lowest level among these methods [83].

In addition to STS, many drugs can induce cell apoptosis through different pathways as mentioned above, and different apoptosis-inducing drugs have been used for different cell types. The use of drugs to induce apoptosis needs to consider the toxicity and other side effects of drugs. UV exposure is a highly effective physical method to promote cell apoptosis. UV exposure is used in radiotherapy of not only malignant tumors but also various inflammatory diseases [84, 85]. This method is suitable for tumor cell apoptosis, but it may lead to mutations in healthy cells. ApoBDs obtained by this method may affect the verification of their therapeutic and regenerative abilities. Starvation, hypoxia, and high fever more closely mimic the normal apoptosis of human cells without the aid of drugs and materials, but there is no specific literature on the comparison of the quantity and quality of ApoBDs produced by cells under these physiological apoptotic conditions. Various methods have been used in apoptosis induction for ApoBDs production in vitro for research, diagnostics, and therapeutic application purpose, but staurosporine (STS) and UV are still the two most commonly used methods (Table 1).

**Table 1:** Methods of apoptosis induction for ApoBDs production in vitro

Method	Specific agent	Process	Cells	Refs.
Chemical	STS	0.5–10 μM STS for 3–12 h	Mesenchymal stem cells, Neutrophils, and human T lymphocytes	[6, 72]
	ALN	500 μM for 24 h at 37 ℃	Bone marrow macrophages, preosteoclasts, and mature osteoclasts	[82]
	Etoposide	10 μM for 24 h	Human T lymphocytes	[83]
	H2O2	1 mmol/L for 12 h	Endplate chondrocyte	[8]
	Actinomycin-D	5 µg/ml for 12 h	Human T lymphocytes	[83]
	TNF-α	100 µM for 12 h	Human T lymphocytes	[83]
Physical	UV exposure	150 mJ/cm^2^ UV for 10 min at a distance of 10 cm	Monocytic leukemia cells, T lymphocytes, promyelocytic leukemia cells, neuronal cells, squamous epithelial cells, and cervical epithelial cells (all from humans)	[33, 73, 74]
Physiological	Serum free culture	Serum starved for 4 h	Monocytic leukemia cells, T cell lymphoma cells, neuronal cells, squamous epithelial cells, and cervical epithelial cells (all from humans)	[33]
	Heat	56 ℃ for 30 min in a water bath	Human promyelocytic leukemia cells	[83]

STS: staurosporine; ALN: azbisphosphonate alendronate

### ApoBDs isolation procedures

**Fig. 4:**
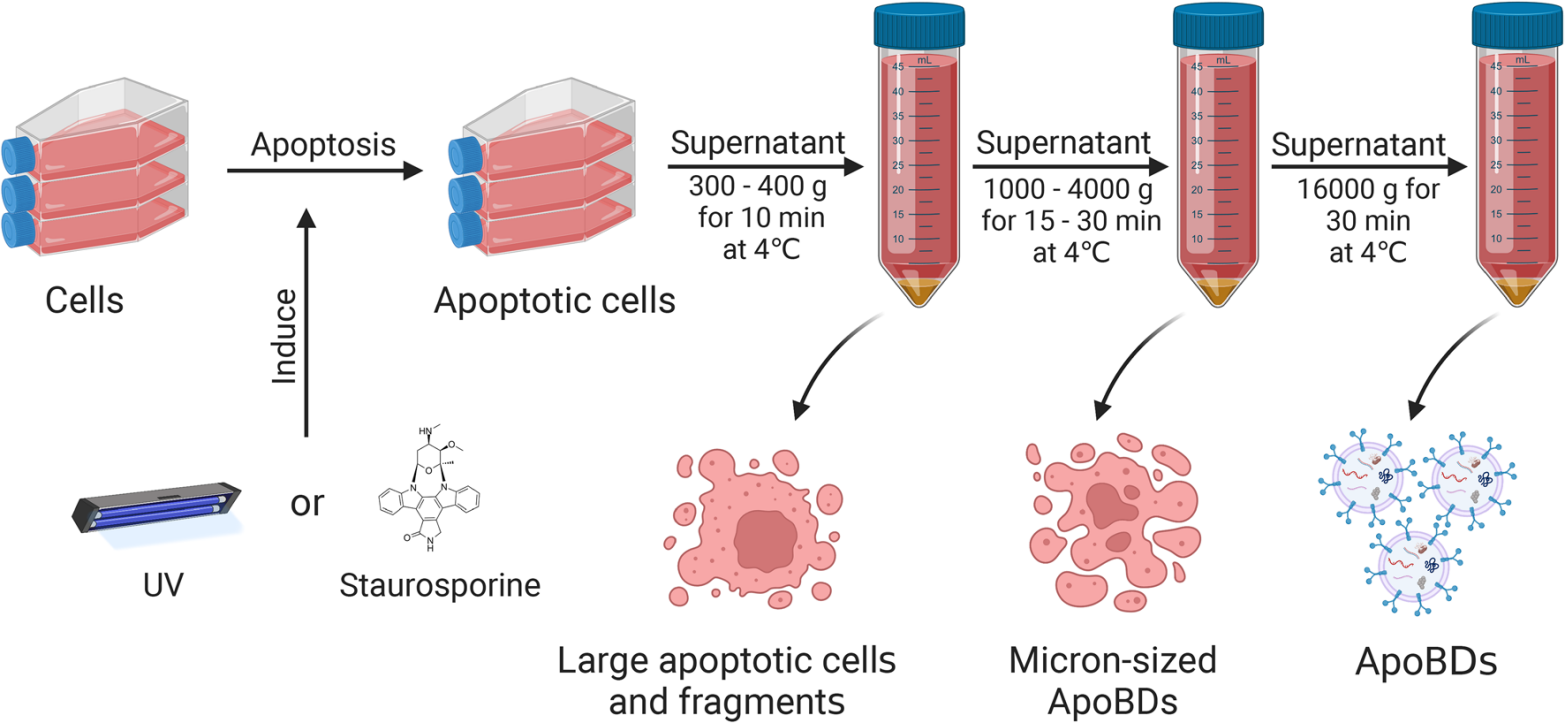
Differential centrifugation for ApoBDs isolation. After induction of apoptosis by UV or staurosporine, the supernatant is centrifuged at 300–400*g* for 10 min at 4 ℃ to remove the large apoptotic cells and fragments. Then, the supernatant is centrifuged at 1000*g*–4000*g* for 15–30 min at 4 ℃ to separate micron-size ApoBDs. At last, nano-size ApoBDs are obtained from precipitate through centrifugation of the supernatant at 16,000*g* for 30 min at 4 ℃. Created with BioRender.com

Differential centrifugation is the gold standard technique for EVs isolation [86]. This method often has a few steps, first, at a low speed of 300–400*g* for 10 min to remove the large apoptotic cells and fragments [87, 88], followed by higher speed spinning (1000*g* to 4000*g*) for 15–30 min at 4 ℃ to separate micron-size ApoBDs which 90% purity could achieve of ApoBDs [9, 89–92]. However, the low rotational speed may lead to the failure of ApoBDs to precipitate and cause a low utilization rate. Some literature used centrifugation at 4 °C at 16,000*g* for 20–30 min after the removal of cells and debris [74, 82, 93]. However, due to these high centrifugal forces, ApoBDs and the smaller microvesicles that can be separated together may affect purity. One limitation of using differential centrifugation for isolating ApoBDs is the co-precipitation of ApoBDs and other EVs, which leads to less sample purity. Because differential centrifugation is used to separate the ApoBDs by density and EVs size, the extracted ApoBDs still contain many impurities. Therefore, many scholars put forward the improved technique of differential centrifugation, such as filtering ApoBDs supernatant obtained from the first centrifugation step (300*g*) and then further centrifugation at 3000*g* [56]. In addition, filtration may lead to artificial fragmentation or lysis of cells or vesicles, so this method should be used with caution (Fig. 4).

Fluorescence-activated cell sorting (FACS) is another method for ApoBDs isolation that can give a higher pure yield of ApoBDs than by differential centrifugation. The purity of ApoBDs prepared by FACS reached 99% [71]. FACS isolates ApoBDs according to their size, PS exposure, and activated PANX1 channels. In addition, cell-type-specific ApoBDs can be isolated with a FACS-based approach including human peripheral blood mononuclear cells, human Jurkat T cells, and primary human umbilical vein endothelial cells [49, 62, 71, 73].

In FACS-based isolation of ApoBDs, the sample is centrifugated at 3000*g* for 6 min yielding 84% of pure ApoBDs [71, 91]. After removing the supernatant, ApoBDs are incubated with A-FITC and TO-Pro-3 in dark for 10 min, and then remove excess stains. Then, the sample is filtered into a flow cytometer with a 70 μm cell filter. At last, the ApoBDs are purified and validated by flow cytometry [91]. The duration of the FACS process depends on the number of ApoBDs. Although the above two methods can extract ApoBDs with high purity, their speed to obtain ApoBDs limits their applied range. Such as size exclusion chromatography, microfluidics, and laser capture approaches of ApoBDs isolation are also worth exploring in the future [56].

## Source and function of various ApoBDs

ApoBDs are produced by the majority of cell types, including cancer cells, stem cells, immune cells, fibroblasts, endothelial cells, and epithelial cells [93–96]. Among them, ApoBDs are characterized by the presence of organelles within the vesicles [38, 97, 98]. Micronuclei, chromatin remnants, cytosol portions, degraded proteins, and fragmented or intact DNA are the main contents of ApoBDs [4, 96]. According to the literature, ApoBDs have various functions including immunomodulation, angiogenesis, and promoting tissue regeneration which can be utilized for the treatment of various diseases [13, 93, 99, 100]. Defects in the clearance of ApoBDs or ApoBDs formation may contribute to the development of autoimmune diseases [101]. So, targeting and clearance of residual ApoBDs is a potential research field in autoimmune disease treatment. In addition, ApoBDs are good carriers for targeted drug delivery due to “eat me” signals and have shown high efficacy in drug delivery to the brain [19, 102]. So, various sources and functions of ApoBDs show great potential in clinical application including diagnosis, disease treatment, and drug delivery.

### Cancer cell-derived ApoBDs

ApoBDs derived from cancer cells have been proven to be closely related to tumor progression [103]. Apoptotic cells affect the signaling capacity of neighboring cells, cell proliferation, tumor growth, angiogenesis, and drug resistance [104–106]. The increased number of ApoBDs is usually found in tumors because macrophages fail to remove apoptotic cells in time. In addition, the phagocytic capacity of macrophages in malignant tumors is also lower than that of healthy macrophages [107]. Aihara et al. found that the number of ApoBDs in the tumor region increased with the degree of malignancy in prostate cancer patients, suggesting that increased programmed cell death is a feature of increased malignant potential [108]. Pavlyukov et al. found that glioblastoma-derived ApoBDs promote tumor growth [109]. Zweemer et al. demonstrated that PS-containing ApoBDs enhance the migration of tumor cells through the PS-GAS6-AXL signaling axis [110]. Based on these reports, we can reasonably speculate cancer cell-derived ApoBDs are important roles in cancer pathophysiology. Approaches for cancer cell-derived ApoBDs deserve more attention and extensive research to establish cancer cell-derived ApoBDs as novel targets for cancer treatment.

Interestingly, cancer cell-derived ApoBDs are not always harmful to our health. Although the ApoBDs of murine melanoma enhanced the risk of thrombotic events, these ApoBDs also showed better effects against cancer through the induction of anti-cancer responses than other extracellular vesicles [111, 112]. Wang et al. achieved good results in the treatment of Parkinson's disease by targeting brain microglial cells with ApoBDs derived from brain metastatic cancer including mouse breast cancer cells, mouse melanoma cells, and immortalized mouse peritoneal macrophages [102]. Bose et al. used ApoBDs from human breast cancer cells, human glioblastoma cells, human hepatocellular carcinoma cells, and mouse breast cancer cells to deliver vancomycin for the treatment of bacterial infections in macrophages and cancer cells [19]. Horrevorts et al. found that melanoma cell lines-derived ApoBDs efficiently deliver drugs and improve the anti-tumor efficacy of the vaccine [113]. Hepatic stellate cells phagocytose HepG2 ApoBDs that induce the survival of the hepatic stellate cells and the spread of liver fibrosis via activation of the JAK1/STAT3 pathway [5]. In addition to directly acting on cells, cancer cell-derived ApoBDs are potential diagnostic indicators of cancers. Since ApoBDs are very common in prostate adenocarcinoma (34%), the presence of ApoBDs should be added to the list of histological features in prostate cancer cases [114]. Reasonable application of cancer cell-derived ApoBDs has great prospects in cancer diagnosis and treatment of various diseases.

### MSCs-derived ApoBDs

MSCs, which can be easily obtained from various tissue types, are multilineage cells with the capability of self-renewal and differentiating into various cell types [115, 116]. MSCs can be obtained from a range of tissues and body fluids, such as dental pulp, periodontal membrane, bone marrow, skin, adipose tissue, and blood [117]. Depending on their origin, MSCs can differentiate into different types of cells that can be used to treat different diseases [118]. Apoptosis of transplanted MSCs is indispensable to the efficacy of mesenchymal stem cell transplantation and mesenchymal stem cell- ApoBDs (MSC-ABs), a type of extracellular vesicles, are produced during this process [119]. In vivo and in vitro studies suggested that MSC-ABs have various properties including immunomodulation, angiogenesis, and cell proliferation, and showed potential in the treatment of several diseases. For example, Liu et al. found that transplanted MSCs enhanced angiogenesis and improved cardiac function recovery by releasing ApoBDs, which revealed the role of donor cell apoptosis in the therapeutic effect of cell transplantation [6]. Liu et al. found that mouse bone marrow-derived mesenchymal stem cells (BMSCs) can trigger the polarization of macrophages toward the M2 phenotype to promote skin wound healing [93]. ApoBDs isolated from mouse MSCs promote skin wound healing and hair regeneration [120]. Ye et al. found that BMSCs-derived ApoBDs after phagocytosis by macrophages reduced COX2 expression in proinflammatory macrophages via the AMPK/SIRT1/NF-κB pathway. In addition, BMSCS-ApoBDs inhibited the secretion of TNF-α and IL-6, increased the secretion of IL-10, and inhibited the formation of adjacent osteoclasts, suggesting that BMSCs can be used in the treatment of periodontitis and bone regeneration [16]. In addition to their regenerative potential, MSCs-derived ApoBDs can be used to treat tumors and blood-related diseases. Wang et al. isolated ApoBDs from mouse BMSCs and then treated multiple myeloma with ApoBDs [14]. Zhang et al. reported that ApoBDs from BMSCs, human adipose tissue-derived MSCs, and mouse BMSCs are more effective in the treatment of mouse hemophilia than exosomes [121]. Therefore, MSCs-derived ApoBDs could be a better alternative to MSCs for the treatment of various diseases.

### Immune cells-derived ApoBDs

The immune system contains a lot of immune cells, such as dendritic cells, T cells, B cells, lymphocytes, macrophages, and so on. The ApoBDs of immune cells have been used in vaccines and drug delivery and obtained good experimental results [122]. ApoBDs derived from immune cells can be better recognized by human immune cells and improve immunogenicity. Zhu et al. found that macrophage-derived ApoBDs can promote the proliferation of receptor cells through microRNA-221/222 [123]. Monocyte-derived phagocytes were more likely to phagocytose ApoBDs derived from lymphoblastic cells containing histones [101]. ApoBDs released by pathogen-infected dendritic cells are internalized by other dendritic cells, which stimulate dendritic cells to present antigens more efficiently [124]. What’s more, autologous T cell ApoBDs can induce the proliferation of peripheral T cells [95]. In addition to improving immunogenicity, ApoBDs can be used for disease diagnosis based on their properties. Isolation and quantification of plasma ApoBDs have been suggested for the evaluation of ischemic stroke and neurodegenerative disease [125]. Atkin-Smith et al. obtained high-purity ApoBDs derived from human Jurkat T cells and primary human umbilical vein endothelial cells by fluorescence-activated cell sorting method [71]. Many studies have shown that ApoBDs are also associated with various diseases, which can be utilized as diagnostic and prognostic tools.

### Other cells-derived ApoBDs

In addition to the apoptotic cells mentioned above, ApoBDs from other cells also have been mentioned in different studies. ApoBDs isolated from an immortalized human vitiligo melanocyte cell line showed that antigen Tyrosinase-related protein 1 (TyrP-1) and cleavage nuclear membrane antigen Lamin A/C (Asp230) are concentrated in ApoBDs, suggesting that ApoBDs may play a key role in the immune destruction of vitiligo [126]. This suggests that ApoBDs can not be a treatment option for other diseases in vitiligo patients. Yuan et al. found that endplate chondrocytes-derived ApoBDs adjust ectonucleotide pyrophosphatase/phosphodiesterase 1 (ENPP1), ankylosis protein (ANK) expression, and tissue‐nonspecific alkaline phosphatase (TNAP) expression to increase PPi metabolism and promote the mineralization of endplate chondrocytes [8]. Osteoclast-derived ApoBDs promote osteogenic differentiation by activating RANKL reverse signaling to stimulate osteoblast differentiation [82]. Non-obese diabetic β-cell-derived ApoBDs reestablish peripheral immune tolerance, and effectively prevent type 1 diabetes mellitus [127]. Tyukavin et al. found that cardiomyocyte-derived ApoBDs stimulate the proliferation and differentiation of resident stem cells both in vitro and in vivo [128]. The biological functions and mechanisms of ApoBDs derived from different cells had been summarized in Table 2.

**Table 2:** Overview of the functions of ApoBDs derived from different cells

Source	Application	Cell type	Function and mechanism	Refs.
Malignant tumor	Drug delivery	Mouse breast metastatic cancer cells	ApoBDs penetrate the blood–brain barrier	[102]
		Human cancer cells (breast, glioblastoma, and hepatic cancer) and mouse breast cancer cells	ApoBDs target macrophages and cancer cells	[19]
		Human melanoma cell	ApoBDs target DCs	[113]
	Therapeutic	Human hepatoma cells	ApoBDs promote survival of HSC via activation of the JAK1/STAT3 pathway	[129]
		Human glioblastoma cell	ApoBDs-mediated transfer of spliceosomal proteins	[109]
		Murine melanoma	Induce anti-tumor immune responses	[111]
	Diagnostic tools	Prostate cancer cells	A higher number of ApoBDs	[108, 114]
MSCs	Therapeutic	Murine MSCs	Promote angiogenesis	[6]
		BMSCs	Reduced COX2 expression	[16]
		Murine BMSCs	Promote M2 macrophage polarization	[6]
	Drug delivery	Human MSCs	ApoBDs-laden hyaluronic acid hydrogel for endometrial regeneration	[74]
Immune cells	Therapeutic	Mouse macrophage	ApoBDs promote the proliferation of recipient epithelial cells	[123]
		Human lymphoblasts	Immunogenic molecules are translocated into ApoBDs	[101]
		DCs	ApoBDs are phagocytosed by other DCs	[124]
		Human T cells	Induce unresponsiveness of peripheral T cells	[95]
		Mouse monocyte	ApoBDs facilitate viral propagation	[7]
	Diagnostic tools	Human plasma	ApoBDs as biomarkers for measuring apoptosis	[125]
Other cells	Therapeutic	Human vitiligo melanocyte	TyrP-1 and Asp230 concentrated in ApoBDs	[126]
		Rat endplate chondrocytes	Adjust ENPP1, ANK, and TNAP expression	[8]
		Osteoclast	Activate RANKL reverse signaling	[82]
		Mouse insulinoma β cells	Reestablish peripheral immune tolerance	[127]
		Rat cardiomyocyte	Stimulate proliferation and differentiation of myocardial cells	[128]

HSC: hepatic stellate cells; MSCs: mesenchymal stem cells; DCs: dendritic cell

## Applications based on non-engineered and engineered ApoBDs

Although ApoBDs is an under-explored EVs in preclinical and clinical settings, ApoBDs have recently been shown to play important roles in physiological homeostasis and pathogenesis. The high yield and purity of ApoBDs are the basis for the clinical application of ApoBDs. As the mechanism of apoptosis becomes more and more clear, the pathway of ApoBDs generation, isolation, purification, and application is also becoming more and more perfect. Because ApoBDs can be rapidly cleared by phagocytes, they are important signaling molecules in tumors, tissue regeneration, and tissue immunity. Based on the current understanding, ApoBDs have been used for diagnostics, vaccines, drug delivery, and treatment of various diseases.

### Applications based on non-engineered ApoBDs

Similar to exosomes, non-engineered ApoBDs show various biological functions including immunomodulation, tumor treatment, and tissue regeneration. In addition, the role of ApoBDs in some diseases and the immunogenicity of ApoBDs make it possible for disease diagnosis or disease vaccines. Recent research indicated that RNA in exosomes can be transferred to acceptor cells and modulate the gene expression in the letter, but the related mechanism and functional molecules of ApoBDs [130]. Different from exosomes, ApoBDs have unique biological functions including apoptotic clearance and modulation of apoptosis-related inflammatory and immune responses [131].

#### Diagnosis and therapeutic potential

As markers of cell death and carriers of dying cell material, ApoBDs have the potential to be valuable evidence of cell death or disease conditions. The application of ApoBDs in disease diagnosis and prognosis, such as malignant tumors, infections, brain injury, and autoimmune diseases, has a good prospect [132]. An increased number of ApoBDs in histological tests for gastrointestinal biopsies indicates gastrointestinal damage [133]. Similarly, it has been reported that ApoBDs have potential in the diagnosis of celiac diseases because the number of ApoBDs in active celiac disease significantly increases [134, 135]. Kawashima et al. found typical ApoBDs in the cerebellar granular layer in Japanese patients with Creutzfeldt-Jakob disease, suggesting that the increase of ApoBDs in the cerebellar granular layer may be important for the disease diagnosis. Interestingly, there was no increase in ApoBDs in cerebellar granulosa in patients with Creutzfeldt-Jakob disease in western countries [136]. Hakan et al. found a large increase in the number of ApoBDs in prostate cancer patients and Eerola et al. found a large number of ApoBDs in the sputum alveolar macrophages of lung cancer patients, which provides a new diagnostic tool for needle biopsy-specific malignancy markers [12, 114]. Drug sensitivity and toxicity of pancreatic tumor cells can also be assessed by impedance cytology of ApoBDs [137]. Some researchers have also proposed the use of crypt ApoBDs markers to detect graft-versus-host disease [135, 138, 139]. It has been suggested that the presence of 6 or fewer crypt ApoBDs is sufficient to diagnose graft-versus-host disease, but there is no conclusive evidence for this diagnosis [138]. Cerebrovascular and neurological lesions may be associated with abnormal apoptosis. The main methods for the detection of apoptotic cells in tissues are invasive procedures. Some scholars put forward the determination of the plasma levels of ApoBDs as the diagnostic tool for apoptosis-related diseases. Therefore, plasma separation and quantitative of ApoBDs is a cost-effective, quick, and minimally invasive procedure [125, 140, 141]. But not all apoptotic diseases differ in the number of ApoBDs, such as the change in the number of cerebrospinal fluid ApoBDs in multiple sclerosis is not significant enough for the diagnosis of the disease [142]. With the deepening of research on ApoBDs and better discovery of biomarkers of ApoBDs-related diseases, ApoBDs could be a rapid, minimally invasive, and accurate diagnostic tool in the future.

#### Vaccine

ApoBDs are also used in vaccines, immunomodulation, drug delivery, and targeted therapy. ApoBDs can improve the immunogenicity of the vaccine, designing a new generation of vaccines for infectious diseases [143]. ApoBDs vaccine products have shown high rates of apoptotic leukemia B cell endocytosis and mass production has begun [144]. The treatment of elderly acute myeloid leukemia by autologous leukemic apoptotic corpse-pulsed dendritic cells has entered the phase I/II clinical study, the study showed that five vaccinated patients survived longer than those who did not receive the vaccine [145]. Hus et al. treated patients with early-stage B-cell chronic lymphocytic leukemia with dendritic cells pulsed with tumor cells derived ApoBDs and achieved satisfactory results [146]. It has also been reported that the ApoBDs of macrophages infected with Leishmaniasis have achieved good results in the treatment of cutaneous leishmaniasis in animal experiments, and these results suggest that ApoBDs are better candidates for vaccine production [147].

#### Immunomodulation

**Fig. 5:**
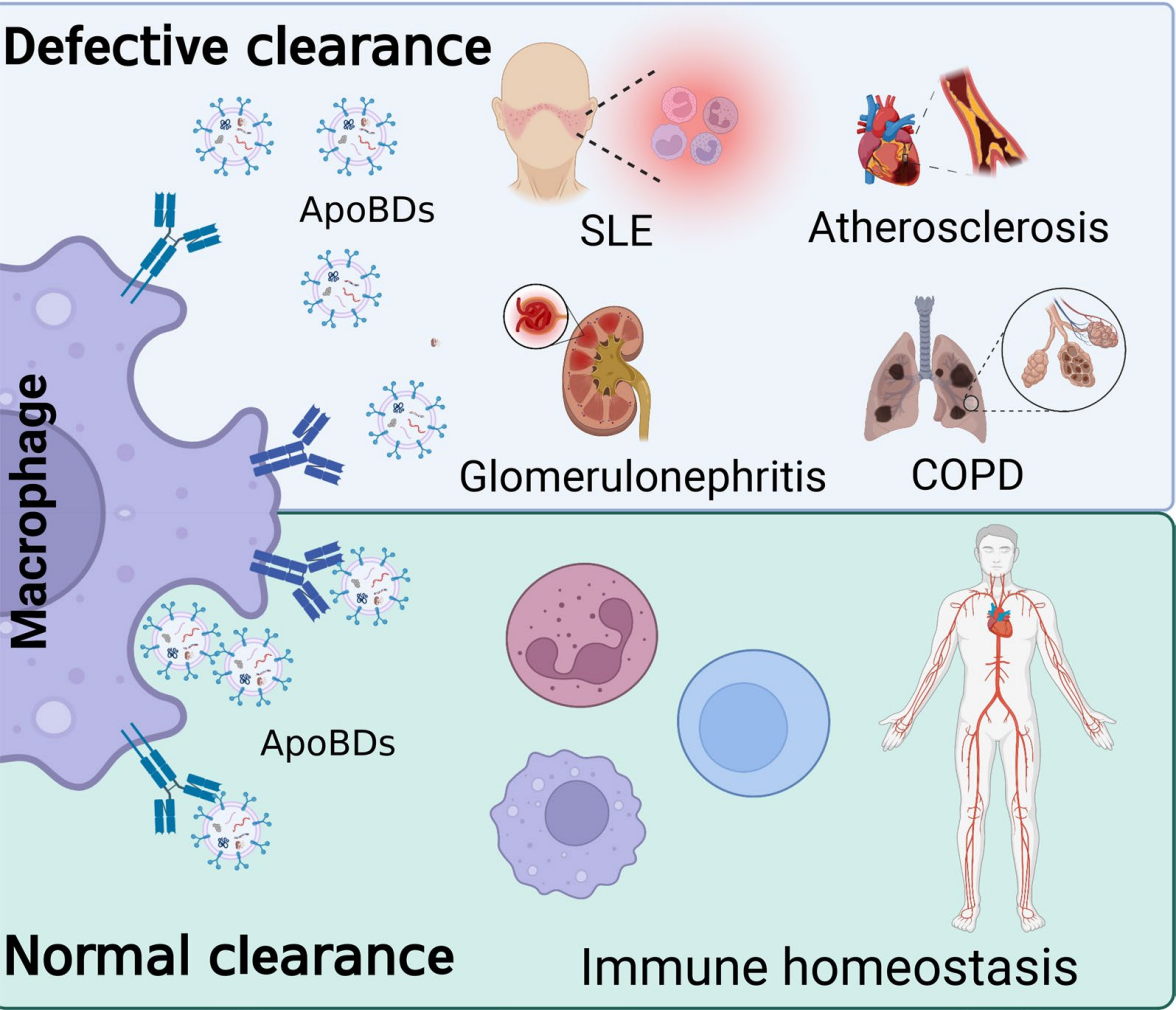
Functional roles of ApoBDs in cell clearance. ApoBDs recruit motile phagocytes by releasing a “find-me” signal to eliminate apoptotic cells and maintain tissue homeostasis. Many systemic diseases, such as SLE, glomerulonephritis, COPD, and atherosclerosis are related to the failure of ApoBDs clearance on time. Created with BioRender.com

During the process of apoptosis, apoptotic cells recruit migratory phagocytes by releasing “find-me” signals and phagocytes recognize apoptotic cells through receptors on phagocytes binding to "eat me" ligands on dying cells. And then intracellular signals are sent within the phagocytes to mediate the internalization of the ApoBDs and the processing of the ingestion [148, 149]. The effective elimination of apoptotic cells is a physiologically critical process that is essential for homeostasis in multicellular organisms. The phagocytosis of apoptotic cells eliminates the debris in the tissue and produces an anti-inflammatory response that inhibits tissue inflammation [150]. Uncleared apoptotic cells can enter a state of pro-inflammatory cell death known as secondary necrosis and trigger inflammatory responses [151]. Impaired cell clearance has been associated with a variety of inflammatory diseases, autoimmune erythematosus, and cardiovascular diseases [152, 153]. At present, ApoBDs are considered new targets to treat inflammatory diseases (Fig. 5).

#### Cancer treatment

Earlier studies had shown that ApoBDs can transfer genetic materials including DNA [154]. Bergsmedh et al. provide evidence that tumor cell-derived ApoBDs transfer tumor DNA horizontally and induce tumor formation in mice [155]. Also, ApoBDs play an important role in tumor immune response. ApoBDs derived from the EL4 T-cell have an immunosuppressive effect on dendritic cells and promote the development of myeloid-derived suppressor cells [156]. ApoBDs derived from irradiated EG7 tumor cells suppress CD8^+^ CTL responses and antitumor immunity through the expression of membrane binding TGF-β1 on the ApoBDs [157]. Interestingly, according to recent evidence, ApoBDs can be immunosuppressive or immunostimulatory in different contexts [158]. Kokhaei et al. indicated that ApoBDs derived from B cell chronic lymphocytic leukemia show a better effect in stimulating T cell response compared with other approaches including tumor cell RNA, tumor cell lysate, fusion hybrids of dendritic cells, and tumor cells [159, 160]. Wang et al. found that ApoBDs activate caspase 3/8 through FasL/Fas pathway to induce apoptosis of multiple myeloma cells [14].

Although it has been proposed that tumor DNA may be transferred horizontally through the uptake of ApoBDs and induce the genetic changes necessary for tumor formation, recent studies have shown that ApoBDs are not significantly associated with tumor progression [103, 161]. In addition, macrophages promote tumor antigen presentation through specific phagocytosis of antigen-presenting cells after ApoBDs in tumors are phagocytosed [162]. Gautier et al. found BRCA1-associated RING domain protein 1 as an autoantigen of ApoBDs-mediated antitumor response, contributing to ApoBDs-mediated antitumor response [163]. These data suggest that the unique immune properties of ApoBDs could provide a valuable tool for cancer immunotherapy. ApoBDs can act as an immune stimulator or immunosuppressor in tumors. This greatly highlights and adds to the complexity of our understanding of how ApoBDs interact with the immune system. Results from in vitro studies may not always reflect exact in vivo conditions, and animal studies are rare. Unfortunately, studies on tumor-derived ApoBDs and their effects on cancer and the immune system are limited. Further studies on the relationship between ApoBDs and tumor progression are mandatory for the clinical application of ApoBDs in cancer therapy.

#### Bone tissue regeneration

A large number of studies have shown that exosomes and microvesicles can promote tissue regeneration. So far, exosomes and microvesicles have been used in studies including nerve, bone, heart, liver, and skin regeneration [164]. Thus, more and more studies have been conducted on the application of ApoBDs in tissue regeneration in recent years.

Bone tissue regeneration is a complex process, which is related to various factors including immunity, inflammatory response, angiogenesis, recruitment of pluripotent stem cells, osteogenic differentiation of stem cells, and bone mineralization [165, 166]. Several studies have reported that ApoBDs induce osteogenic differentiation, reverse MSC injury, and inhibit M1 polarization of macrophage, which indicated the potential of ApoBDs in bone tissue regeneration. Landry et al. found that the number of osteoblasts and ApoBDs reached a peak in the process of bone injury healing, suggesting that apoptosis may be related to bone healing [167]. Olmedo et al. demonstrated that adding cytokines after bone injury in mice did not affect either the proliferation of osteoprogenitor cells or the concentration of osteoblasts, suggesting that the activation of apoptosis during injury repair is not necessarily a passive result of cell response to injury. Programmed cell death may play an active role in regulating bone repair [168]. Ma et al. found that osteoclast-derived ApoBDs stimulate osteogenic differentiation by activating RANKL reverse signaling. The osteogenic ability of ApoBDs was the strongest in EVs, suggesting that osteoclasts may regulate their differentiation by influencing osteoclast precursor differentiation through ApoBDs production [82]. More importantly, in vitro and in vivo studies have shown functional and proteomic similarities of ApoBDs to their parental cells [130]. Liu et al. demonstrated that systemic injection of ApoBDs derived from exogenous BMSCS reversed MSC injury and improved osteopenia in ovariectomized mice, confirming the role of ApoBDs in maintaining bone homeostasis and demonstrating the potential of ApoBDs in the treatment of osteoporosis. Ye et al. found BMSCs-derived ApoBDs inhibit the polarization of macrophages into a pro-inflammatory state through AMPK/SIRT1/NF-κB pathway, and inhibit the secretion of TNF-α from the pro-inflammatory macrophages to inhibit the formation of adjacent osteoclasts [16].

#### Soft tissue regeneration

**Fig. 6:**
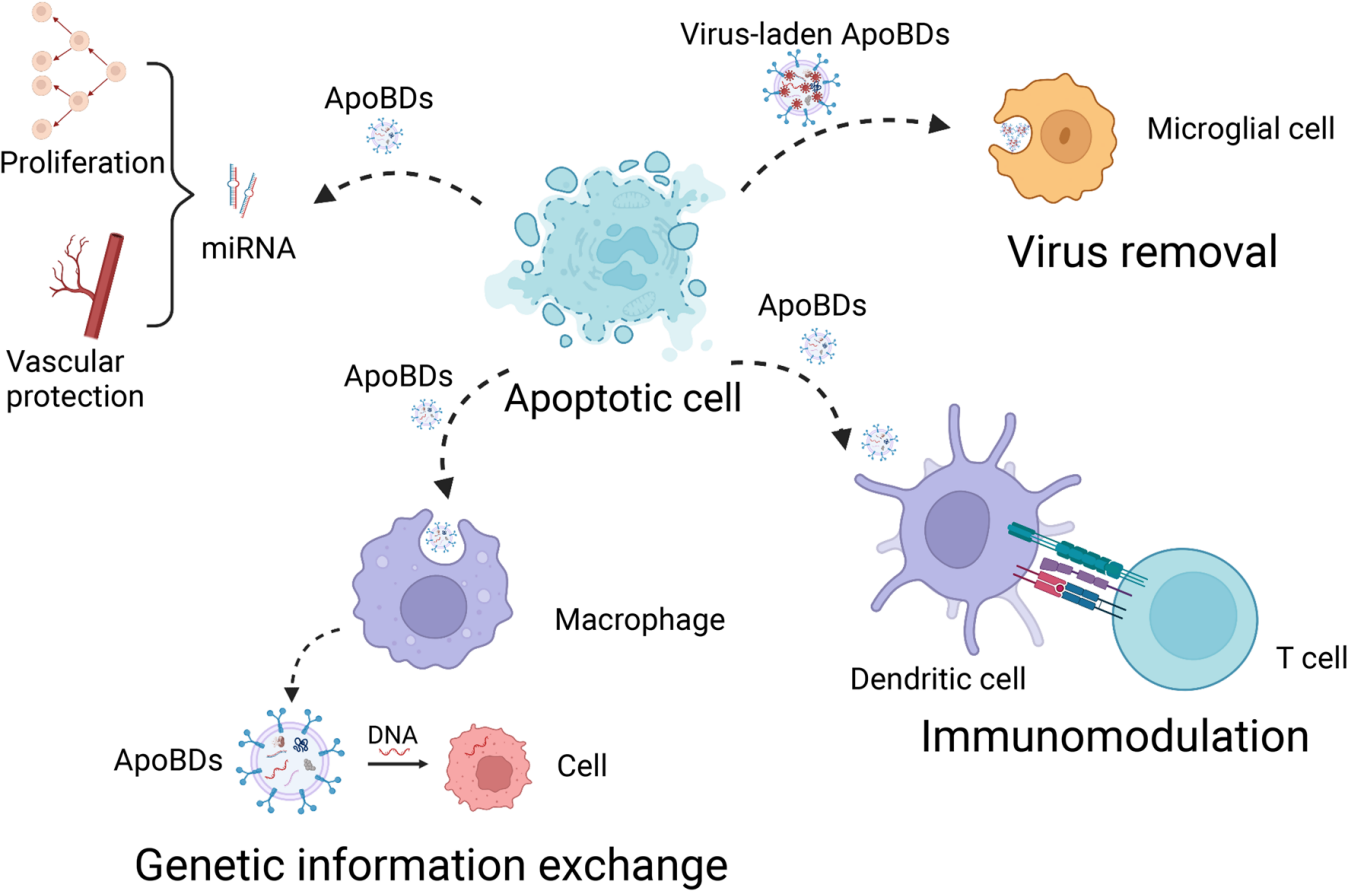
Functional roles of ApoBDs in intercellular communication. ApoBDs can bind to a variety of receptor cells to facilitate intercellular communication and promote proliferation, vascular protection, virus removal, genetic information exchange, and immunomodulation through signaling molecules. Created with BioRender.com

Cardiovascular diseases, including heart disease, hypertension, and arrhythmias, are major diseases that affect human health and survival, and more than 130 million adults in America are projected to have cardiovascular diseases by 2035 [169, 170]. As a controversial research, various strategies were proposed to repair damaged cardiovascular diseases, including stem cell transplantation, stimulating cardiac myocyte proliferation, and differentiation [171]. Hristov et al. found that ApoBDs of endothelial cells promote the repair of damaged endothelium by promoting the proliferation and differentiation of endothelial progenitor cells [172]. Zernecke et al. found that Mir-126-carrying human endothelial cell-derived ApoBDs up-regulate CXCL12 in vascular cells, which recruit the incorporation of hematopoietic stem cells to promote vascular protection and inhibit atherosclerosis [173]. Tyukavin et al. found that ApoBDs of cardiomyocytes stimulated the proliferation and differentiation of cardiomyocytes in mice [128]. In addition, ApoBDs of cardiomyocytes and fibroblasts stimulate stem cells in the heart. ApoBDs of cardiomyocytes promote clones of cardiomyocyte precursors in the myocardium, while ApoBDs of fibroblasts stimulate the formation of endothelial precursor clones [174]. Liu et al. found that MSCs-derived ApoBDs enhance lysosome biosynthesis and regulate autophagy and angiogenesis in recipient endothelial cells by activating transcription factor EB. Thus, mesenchymal stem cells ApoBDs can promote angiogenesis and cardiac function recovery after myocardial infarction [6]. ApoBDs can also be used for drug delivery and targeted therapy of myocardial infarction. These studies highlight the importance of the intercellular communication role of ApoBDs in physiological and pathological conditions (Fig. 6).

**Fig. 7:**
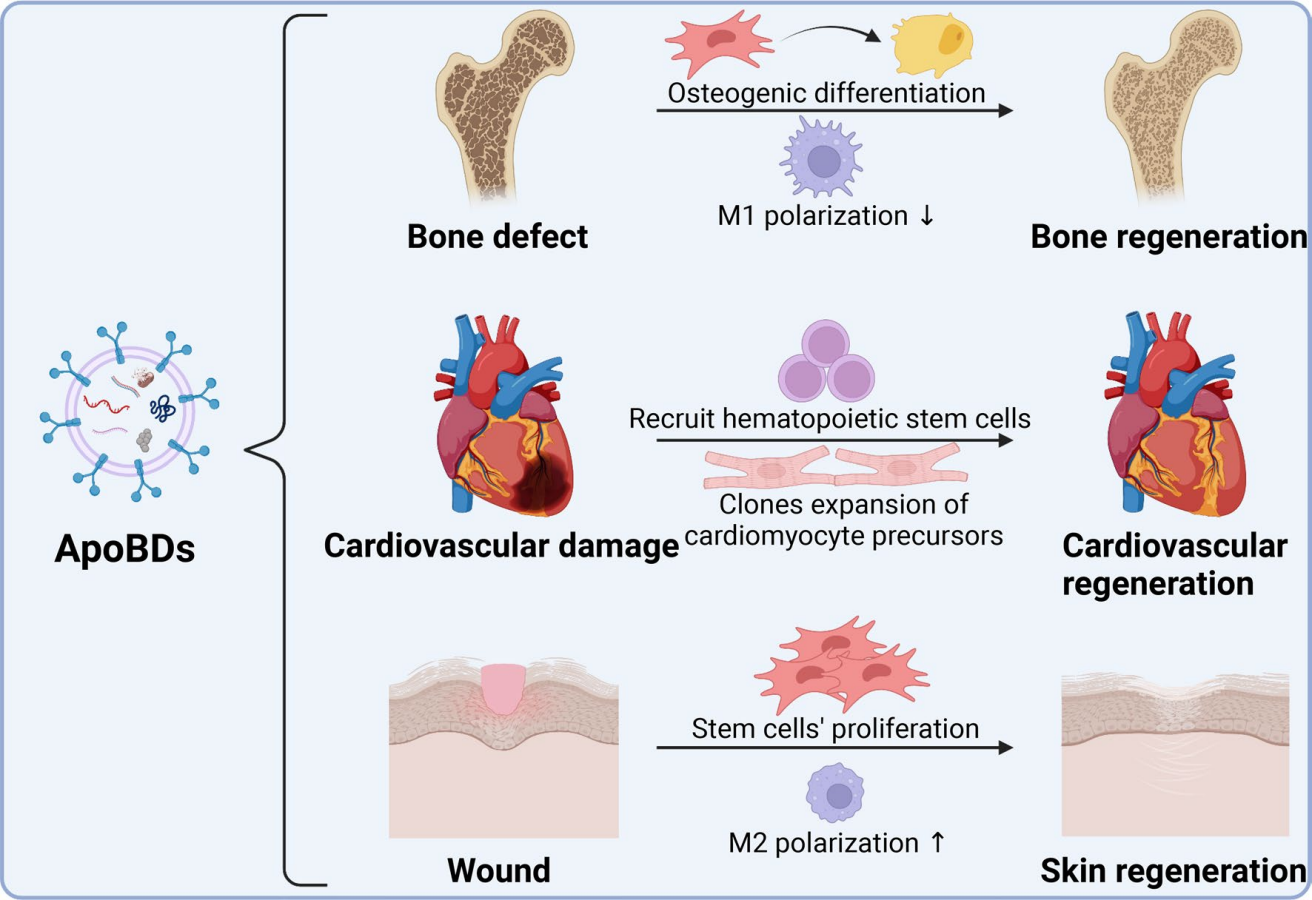
ApoBDs have the potential to promote tissue regeneration. Bone regeneration: ApoBDs promote bone regeneration by stimulating osteoclast differentiation and inhibiting the M1 polarization of macrophages. Cardiovascular regeneration: ApoBDs of fibroblasts stimulate the formation of endothelial precursor clones and ApoBDs of cardiomyocytes promote clones of cardiomyocyte precursors. Skin regeneration: ApoBDs promote the proliferation of stem cells and the M2 polarization of macrophages. Created with BioRender.com

**Fig. 8:**
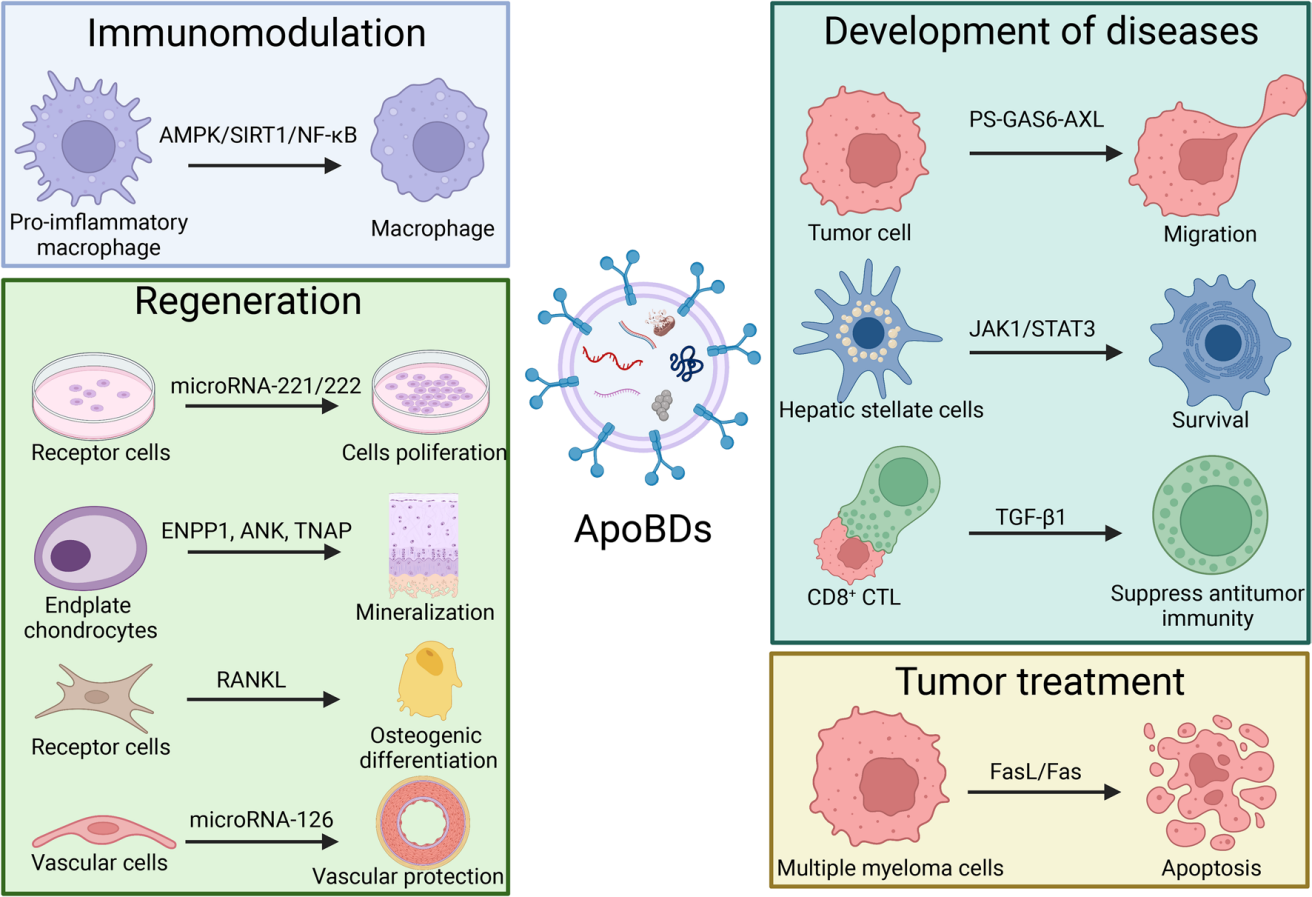
The biological mechanism and functional molecules of ApoBDs. ApoBDs exert various biological functions by modulating different signal pathways and transferring miRNA and cytokines

Dying stem cells promote communication with neighboring stem cells through caspase-dependent production of ApoBDs containing Wnt8α to promote cell transformation of living epithelial cells, which indicates that ApoBDs have potential in epithelial tissue maintenance and repair through stimulating the proliferation of healthy stem cells [175]. In addition, ApoBDs of mesenchymal stem cells promote the polarization of macrophages to the M2 phenotype and play a key role in tissue repair, and promote skin wound healing [93]. Ma et al. found that ApoBDs can promote skin wound healing and hair regeneration by activating the Wnt/β-catenin pathway [120]. In mouse experiments, caspase 3 and 7 cleave and activate iPLA2, eventually triggering the production of growth signal PGE2, and promoting epithelial wound healing and liver tissue regeneration [176]. Proteolytic inactivation of caspase 3 in dying tumor cells attenuates angiogenic effects in vitro and in vivo. Caspase 3 in dying tumor cells establishes an angiogenic microenvironment and drives angiogenic effects [177, 178]. Chera et al. found that decapitated hydra regenerates their heads through morphallaxis, which occurred through apoptosis-inducing Wnt3 [179, 180]. Further experiments showed that the transient release of Wnt3 by apoptotic cells induces MAPK-dependent activation of compensatory proliferation [181]. Brock et al. demonstrated that basal stem cell ApoBDs activate Wnt8a signaling to promote stem cell proliferation and maintain epithelial regeneration and homeostasis (Fig. 7) [175]. Phagocytosis of ApoBDs promotes hepatic stellate cell survival through JAK1/STAT3 and PI3K/Akt/NF-κB pathways [129]. The critical functional molecules and the related mechanism of ApoBDs were summarized in Fig. 8.

### Application based on engineered ApoBDs

Recent studies indicated that ApoBDs have the potential for cancer treatment, immunomodulation, and infection treatment. To enhance the original therapeutic effect of ApoBDs, various engineered ApoBDs have been developed, including functional modification, drug loading, and drug combination.

#### Cancer treatment

Zhao et al. achieved deep tumor penetration through the ApoBDs-mediated proximity effect, proving that ApoBDs can carry the remaining drugs to adjacent tumor cells [182]. It revealed the great potential of ApoBDs for drug delivery and targeted therapy. MMP2-sensitive apoptotic body-mimicking nanoparticles are effective in identifying tumor-specific macrophages through the "eat me" signal in the apoptosis pathway in various biological models. So, ApoBDs as tumor-targeting delivery systems have great potential in the diagnosis and treatment of cancer targeting tumor-associated macrophages [183]. Boisteau et al. found that immunotherapy using ApoBDs in combination with IL2 in colon cancer tumor-bearing mice showed a 50% improvement in tumor remission and survival in vaccinated rats [184]. Muhsin-Sharafaldine et al. demonstrated that ApoBDs are more effective than exosomes in promoting blood clotting, and PS in ApoBDs is crucial in promoting blood clotting activity. Antigen-pulsed ApoBDs are used to protect B16 melanoma tumor-attacked mice for 60 days, and the results indicated a higher protection rate of ApoBDs against tumors compared with other EVs [111]. In B-cell chronic lymphocytic leukemia patients, T cells stimulated with tumor-derived ApoBDs-pretreated DCs secrete high levels of IFN-γ and improve clinical efficacy [159].

#### Immunomodulation

Gou et al. found that cytarabine-induced ApoBDs encapsulate IGF2BP3 and promote the survival of recipient cells by activating PI3K-AKT and p42-44 MAPK pathways. It provides new ideas for drug resistance to myelodysplastic syndrome and adult acute myeloid leukemia [185]. Gallen et al. found that non-obese diabetic β-cell-derived ApoBDs pulsed dendritic cells reduce the expression of costimulatory molecules CD40, CD86, and DC proinflammatory cytokines to reestablish peripheral immune tolerance that effectively prevents type 1 diabetes mellitus [127]. Wu et al. constructed apoptotic biomimetic liposomes for the selective delivery of pioglitazone into atherosclerotic macrophages. These novel liposomes can effectively target atherosclerotic plaques and prevent the progression of atherosclerosis by upregulating the number of anti-inflammatory macrophages and stabilizing atherosclerotic plaques [18]. Bao et al. used ApoBDs loaded with stimulators of interferon genes to activate and improve tumor immunogenicity and achieved good results [186]. Recently, some scholars reported that ApoBDs loaded with anti-TNF-α antisense oligonucleotide can penetrate the blood–brain barrier and ultimately be absorbed by microglia in the brain, which significantly mitigates Parkinson's disease [102]. Dou et al. constructed chimeric ApoBDs for on-demand modulation by combining pure membrane from ApoBDs as a bioconjugation/regulation module and mesoporous silica nanoparticles as a carrier module [187]. Chimeric ApoBDs loaded with microRNA-21 or curcumin target macrophages in inflammatory regions, effectively promoting the M2 polarization to regulate inflammation, and alleviate inflammatory bowel disease in mouse models. Type 1 diabetes is characterized by an autoimmune attack on insulin-producing beta cells. Marin-Gallen et al. reported that pulsed with β cell-derived ApoBDs significantly reduced the incidence of diabetes and islet inflammation and type 1 diabetes mellitus recurrence in accelerated autoimmune diabetic mice dendritic cells [127]. Yan et al. constructed engineered neutrophil ApoBDs with natural neutrophil ApoBDs membrane and 5-aminolevulpionate hexyl esterate (HAL) to simulate natural neutrophil apoptosis. In vivo studies showed that engineered neutrophil ApoBDs can effectively regulate the inflammatory response in the infarct area to improve cardiac function and promote the repair of myocardial infarction [72]. Engineered neutrophil ApoBDs have an excellent ability to target inflamed tissue and control inflammation in areas of myocardial injury using a novel cell-free therapy [72].

#### Treatment of infection

Whiteside et al. use ApoBDs as vectors for the treatment of HIV infection which have shown good results in cell and animal studies and have initiated clinical trials demonstrating that ApoBDs are safe and effective for drug delivery [188, 189]. Plasma ApoBDs derived from patients with acute HIV-1 infection inhibit cytokines production of dendritic cells possibly through an unclear mechanism of CD44, resulting in subsequent perturbed Th1 and NK cell response [190]. The recombinant ApoBDs extracted from cancer cells have been used in the treatment of refractory infections as "nano bait" by targeting the inherent "eat me" signal of ApoBDs and have achieved good results. In vivo and in vitro studies have shown that vancomycin-loaded recombinant ApoBDs are more effective in treating Staphylococcus aureus-infected macrophages than vancomycin-only [19].

**Fig. 9:**
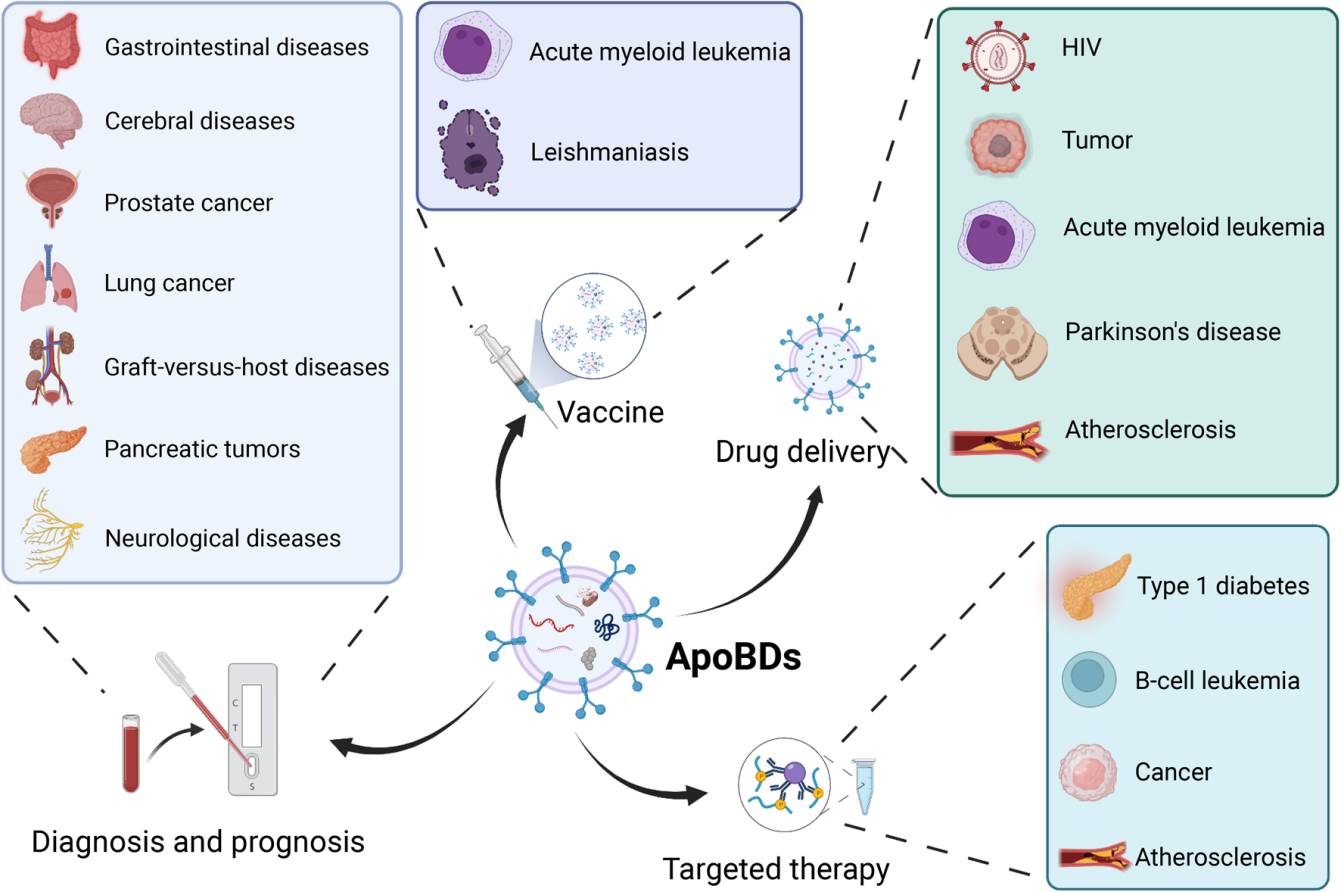
Application of ApoBDs in various diseases. As markers of cell death and carriers of dying cell material, ApoBDs have great potential to apply in disease diagnosis/prognosis, vaccines, immunomodulation, drug delivery, and targeted therapy. Created with BioRender.com

Besides, engineered ApoBDs have also been used in tissue regeneration. Xin et al. found that injection of the ApoBDs laden with hyaluronic acid could efficiently reduce fibrosis and promote fibrosis endometrial regeneration, resulting in fertility restoration [74]. These reports demonstrated that ApoBDs are excellent vectors for targeted therapy because they not only have extraordinary delivery efficiency but also have a higher potential for mass production than cell-based other vectors. The potential clinical application of ApoBDs had been summarized in Fig. 9.

## Challenges and prospects

**Fig. 10:**
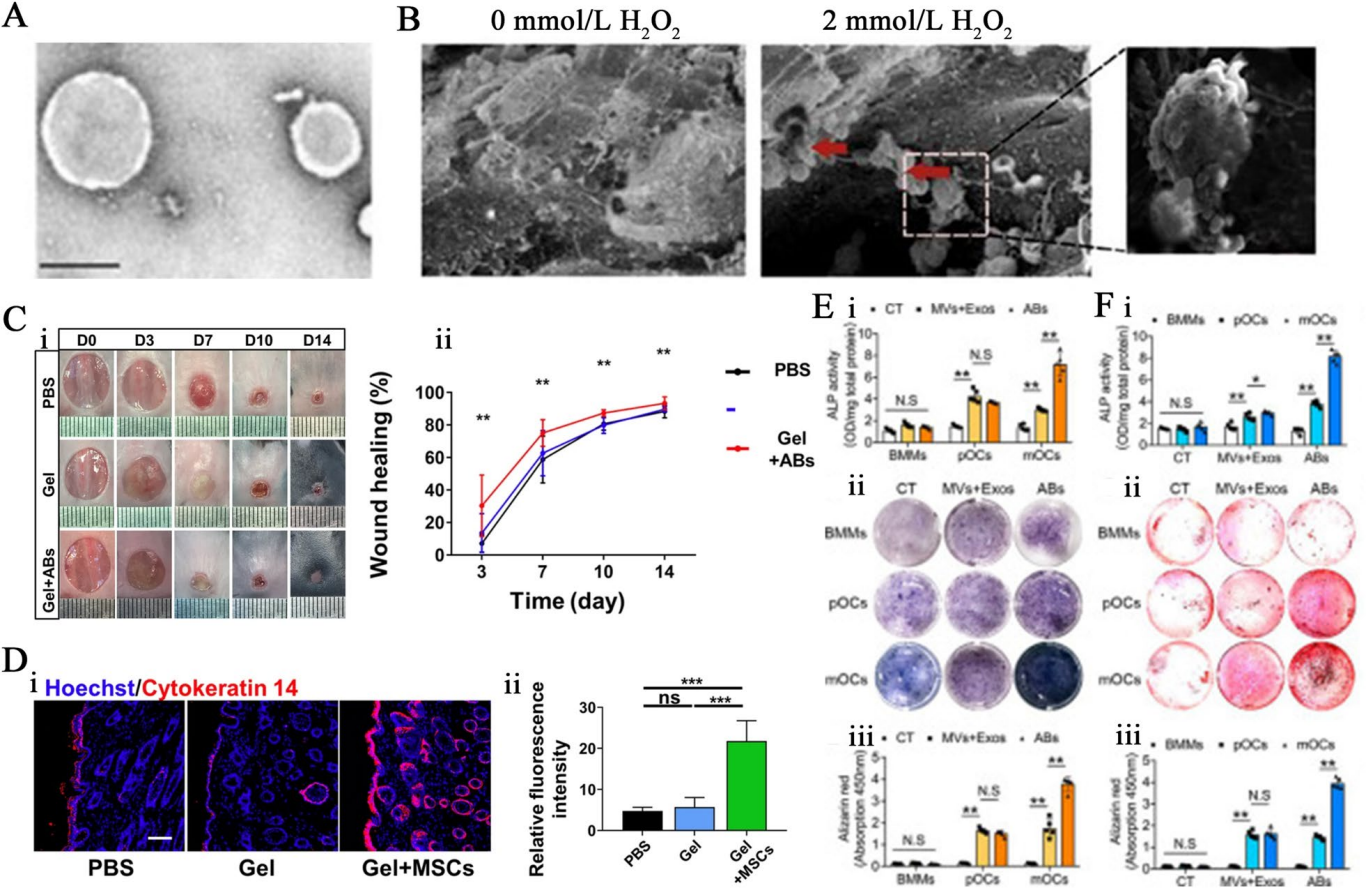
Therapeutic potential of ApoBDs in tissue regeneration, inflammation modulation, and cancer treatment. **A** Transmission electron microscopy image showing the morphology of ApoBDs. Source: Reprinted with permission from Ref. (99). **B** Scanning electron microscopy image of the presence of ApoBDs from H2O2-treated cells. Source: Reprinted with permission from Ref. (8). Copyright 2019, with permission from Creative Commons CC BY. **C** ApoBDs promoted cutaneous wound healing. **Ci** Photographs of cutaneous wounds during the wound healing procedure. **Cii** Quantification of the wound healing rate. Source: Reprinted with permission from Ref. (66). Copyright 2020, with permission from Creative Commons CC BY. **D** Images (**Di**) and quantification (**Dii**) of the cytokeratin 14 expression in the skin tissue. Source: Reprinted with permission from Ref. (66). Copyright 2020, with permission from Creative Commons CC BY. **E**, **F** Mature osteoclast-derived ApoBDs have the highest osteogenic potency in ApoBDs, microvesicles, and exosomes from mature osteoclast, bone marrow macrophage, and preosteoclast. ALP activity (**Ei** and Fi), ALP stain (**Eii**), Alizarin red (**Fii**), and quantification of Alizarin Red activity (**Eiii** and **Fiii**). Source: Reprinted with permission from Ref. (79 Copyright 2019, with permission from Creative Commons CC BY

ApoBDs can originate from a variety of cells. They are vesicles of information and material from dying cells and were previously regarded as apoptotic debris until they were found to be able to deliver useful material to healthy recipient cells [97]. The formation of ApoBDs promotes the effective removal of cell debris through the surrounding phagocytes, thereby preventing secondary necrosis and maintaining immune homeostasis. Failure to remove apoptotic debris can lead to inflammation or autoimmune disease [151]. ApoBDs are the apoptotic cells' residual material and play an important role in intercellular communication by transporting signaling molecules, just like exosomes and microvesicles. In recent years, numerous reports have shown the great potential of ApoBDs inflammatory disease treatment, immune regulation, drug delivery, disease diagnosis, targeted therapy, and tissue regeneration. ApoBDs’ biologically active molecules, including proteins, mRNAs and miRNAs, and DNA transport genetic information from one cell to another, leading to the exchange of genetic information and the reprogramming of recipient cells [161]. For example, the DNA contained in lymphoma-derived ApoBDs is engulfed by surrounding fibroblasts, which results in the integration of lymphoma-derived DNA into the fibroblast genome [161]. Some studies indicated that ApoBDs have procoagulant and immunogenic effects, suggesting the potential of ApoBDs to promote the prethrombotic state and anticancer immunity [83, 111, 112]. ApoBDs homeostasis is associated with a variety of diseases and defective clearance of ApoBDs can lead to prolonged inflammatory responses, autoimmune diseases, and other hazards [191]. Apoptotic cell clearance becomes less efficient with aging, but the mechanism of how aging affects apoptotic cell clearance remains unknown [191, 192]. On the other hand, recently reports indicated that ApoBDs have been shown to promote tissue regeneration, such as bone regeneration, cardiovascular regeneration, skin regeneration, and so on. According to these reports, the structure, size and occurrence can be observed through TEM and SEM, and biological functions of ApoBDs can be detected through immunofluorescence staining, ALP staining, Alizarin red staining, and cutaneous wound healing test (Fig. 10). ApoBDs may be an effective and safe treatment in cell product-based cell-free regenerative medicine and may be a suitable alternative to conventional drug therapy and stem cell therapy.

Although ApoBDs have great potential in the diagnosis and therapy of various diseases, there are still some challenges in the clinical application of ApoBDs. Firstly, it is necessary to explore a standardized procedure to produce ApoBDs for clinical use. The formation of ApoBDs is the result of cell disintegration, which is a complex process of programmed death of organisms. Depending on the mechanism used by the particular cell type undergoing apoptotic cell breakdown, different quantities and qualities of ApoBDs will be produced. The heterogeneity, storage conditions, quality control, standardized separation, and purification process should be addressed. Secondly, the targeting and phagocytosis mechanisms of ApoBDs need more investigation. Several molecules have been proposed as signals of phagocytosis, including PS, calreticulin, and other signals on the surface of apoptotic cells. However, the exact mechanism is still controversial. Further studies on the targeting, classification, and phagocytosis mechanisms of ApoBDs will magnify their therapeutic effects and overcome these limitations, which will become one of the key points for the breakthrough of ApoBDs research. Thirdly, the standards of using ApoBDs as diagnostic approaches need further research. ApoBDs have great potential as a rapid, accurate, and minimally invasive diagnosis. However, the standards of the number and quality of ApoBDs detected, detection approaches, detection instruments, and the necessary skills of personnel are still unclear. Moreover, the relationship between disease and ApoBDs is still unclear.

**Fig. 11:**
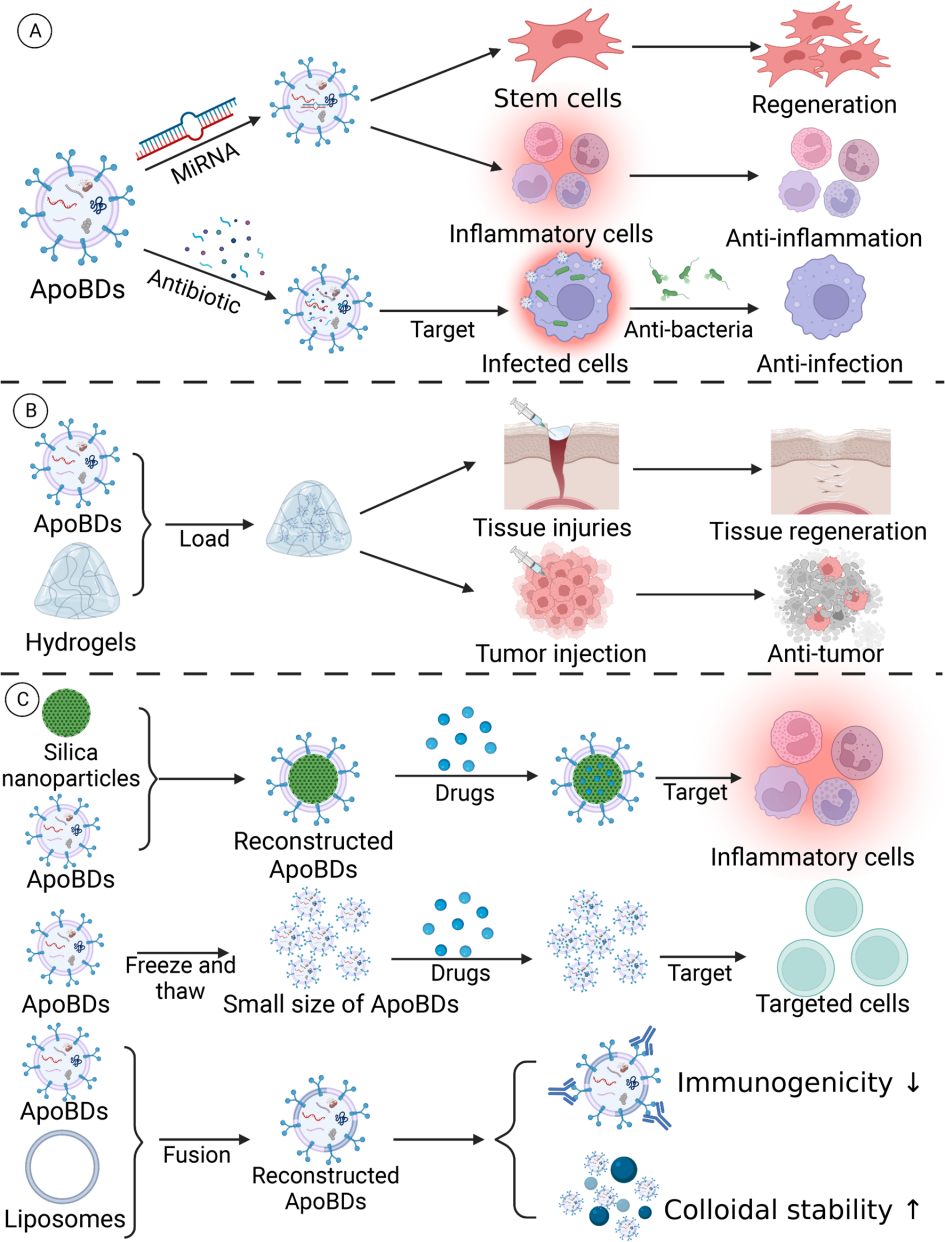
The possible approaches for modifications of ApoBDs for therapeutic applications. **A** ApoBDs have great potential in regeneration, anti-inflammation, and anti-infection, through drug delivery ability. **B** Hydrogel can be used for sustained release of ApoBDs, which has promising capability in tissue regeneration and anti-tumor therapy. **C** Several methods have been explored to induce the drug delivery ability, physical properties, and biocompatibility of ApoBDs, such as constructed with inorganic nanoparticles, freeze and thaw, and membrane fusion with liposome. Created with BioRender.com

ApoBDs have great potential in immune regulation, diagnosis, infection treatment, and cancer treatment due to their drug delivery ability, special biogenesis, and therapeutic effects including anti-inflammation, regenerative, procoagulant, and immunogenic effects. As a cell-free therapy, ApoBDs have fewer ethical and biological issues than stem cell transplantation, such as aberrant differentiation and stress-induced necrosis [193]. To enhance the therapeutic capability of EVs, multiple strategies have been used in modifying EVs, such as genetic manipulation, metabolic labeling, cellular uptake of exogenous material, active loading, and covalent/non-covalent modification of EVs membrane [193]. Based on the drug delivery and targeting ability of ApoBDs, ApoBDs can carry and deliver DNA, RNA, antisense oligonucleotides, antibiotics, or other hydrophobic, hydrophobic, and lipophilic drugs, which improve the efficacy of drugs and ApoBDs [19, 102, 194, 195]. In addition, ApoBDs have the potential for disease treatment and tissue regeneration combined with biological matrices and scaffolds such as hydrogels and thermal, similar to other nanoparticles or stem cells [196–200]. Besides, due to the special “eat-me” signals on the surface of ApoBDs, constructing engineered exogenous vesicles coated with the membrane of ApoBDs is a feasible method to obtain an ideal drug carrier with good shape and targeting function. Dou et al. constructed chimeric ApoBDs with ApoBD’s membrane and mesoporous silica nanoparticles to deliver drugs to macrophages in the inflammatory region [187]. In addition, the freeze and thaw process reconstructs ApoBDs of the desired size. Rajendran et al. reported that they successfully reduced the size of ApoBDs from 1–10 μm to 100–150 nm through this process [19]. It has been proved that membrane fusion between exosomes and liposomes changes the surface of exosomes [201]. Membrane fusion between synthetic liposomes and ApoBDs is a possible way to modify the surface of ApoBDs to decrease immunogenicity and increase the colloidal stability of ApoBDs. Therefore, ApoBDs have a broad prospect in therapeutic applications (Fig. 11).

**Fig. 12:**
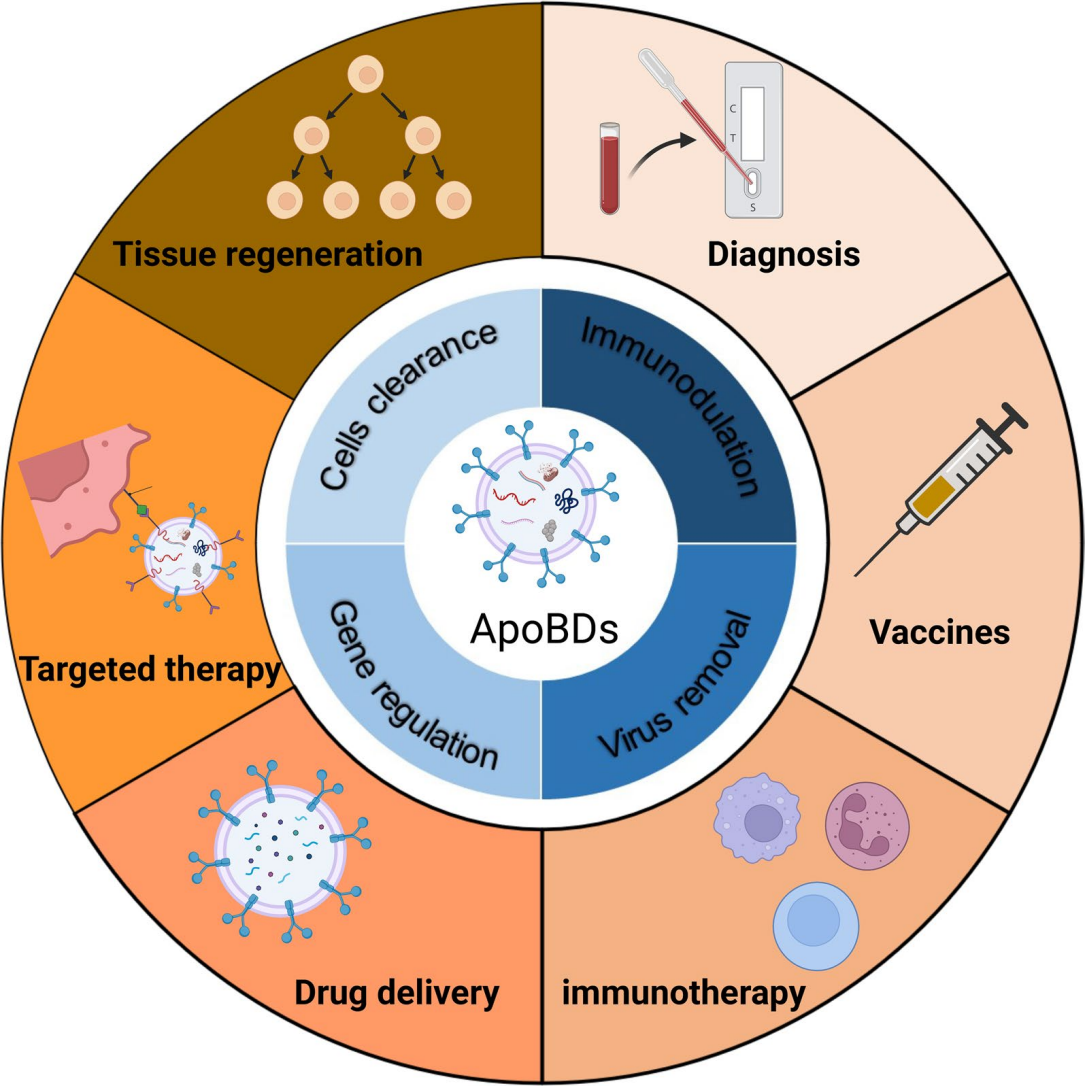
Advances and prospects of ApoBDs in diagnostic and therapeutic applications. Created with BioRender.com

ApoBDs play a regulatory role in a variety of physiological and pathological processes. Optimizing the induction of the cell apoptosis process in vitro, the quality and quantity of ApoBDs, drug loading efficiency, and targeted therapy approach could provide novel prospects in the ApoBDs-based treatment of various diseases (Fig. 12). However, further studies are needed to understand the mechanisms of cell type-specific internalization of ApoBDs during the treatment of different diseases.

## Conclusion

In conclusion, ApoBDs are more than just cell debris left behind by dying cells. According to recent discoveries, ApoBDs are emerging as bioactive treasures left behind by dying cells with an important role in intercellular communication with various biological functions including immunomodulation, cell clearance, gene regulation, and virus removal. These unique biological functions indicate the promising application potential of ApoBDs in diagnosis, vaccine development, immunomodulation, drug delivery, targeted therapy, cancer therapy, and tissue regeneration. Besides the great achievements in the field of ApoBDs, there are still a lot of challenges for the clinical application of ApoBDs. The therapeutic components present in the ApoBDs and the mechanism of ApoBDs-mediated effects in target cells are still not fully clarified. Based on the reports from recent literature, ApoBDs can be predicted as a new player in the field of disease diagnosis, prognosis, and treatment.

## Data Availability

Data sharing not applicable to this article as no datasets were generated or analysed during the current study.

## Abbreviations

ApoBDs: Apoptotic bodies
EVs: Extracellular vesicles
PS: Phosphatidylserine
SLE: Systemic lupus erythematosus
NO: Nitric oxide
DCs: Dendritic cells
MSC: Mesenchymal stem cell
APAF1: Apoptotic peptidase activating factor 1
DISC: Death-inducing signaling complex
AVD: Apoptotic volume decrease
PAK2: P21 activated kinase 2
LIMK1: Lim domain kinase 1
ROCK1: Rho-associated kinase 1
PANX1: Pannexin 1
STS: Staurosporine
BMSCs: Bone marrow-derived mesenchymal stem cells
TyrP-1: Tyrosinase-related protein 1
Asp230: Cleavage nuclear membrane antigen Lamin A/C
ENPP1: Ectonucleotide pyrophosphatase/phosphodiesterase 1
ANK: Ankylosis protein
TNAP: Tissue‐nonspecific alkaline phosphatase
FACS: Fluorescence-activated cell sorting
COPD: Chronic obstructive pulmonary disease
HIV-1: Human immunodeficiency virus-1
